# The Integrated Landscape of Biological Candidate Causal Genes in Coronary Artery Disease

**DOI:** 10.3389/fgene.2020.00320

**Published:** 2020-04-21

**Authors:** Qiwen Zheng, Yujia Ma, Si Chen, Qianzi Che, Dafang Chen

**Affiliations:** Department of Epidemiology and Biostatistics, School of Public Health, Peking University, Beijing, China

**Keywords:** coronary artery disease, genome-wide association studies, prioritize, causal genes, expression quantitative trait locus, protein–protein interaction, network, integration analysis

## Abstract

**Background:**

Genome-wide association studies (GWASs) have identified more than 150 genetic loci that demonstrate robust association with coronary artery disease (CAD). In contrast to the success of GWAS, the translation from statistical signals to biological mechanism and exploration of causal genes for drug development remain difficult, owing to the complexity of gene regulatory and linkage disequilibrium patterns. We aim to prioritize the plausible causal genes for CAD at a genome-wide level.

**Methods:**

We integrated the latest GWAS summary statistics with other omics data from different layers and utilized eight different computational methods to predict CAD potential causal genes. The prioritized candidate genes were further characterized by pathway enrichment analysis, tissue-specific expression analysis, and pathway crosstalk analysis.

**Results:**

Our analysis identified 55 high-confidence causal genes for CAD, among which 15 genes (*LPL*, *COL4A2*, *PLG*, *CDKN2B*, *COL4A1*, *FES*, *FLT1*, *FN1*, *IL6R*, *LPA*, *PCSK9*, *PSRC1*, *SMAD3*, *SWAP70*, and *VAMP8*) ranked the highest priority because of consistent evidence from different data-driven approaches. GO analysis showed that these plausible causal genes were enriched in lipid metabolic and extracellular regions. Tissue-specific enrichment analysis revealed that these genes were significantly overexpressed in adipose and liver tissues. Further, KEGG and crosstalk analysis also revealed several key pathways involved in the pathogenesis of CAD.

**Conclusion:**

Our study delineated the landscape of CAD potential causal genes and highlighted several biological processes involved in CAD pathogenesis. Further studies and experimental validations of these genes may shed light on mechanistic insights into CAD development and provide potential drug targets for future therapeutics.

## Highlights

- Conducted an integrative analysis to prioritize the CAD potential causal genes by using eight computational methods.
- Identified 55 high-confidence causal genes for CAD, among which 15 genes ranked the highest priority.
- Plausible causal genes were enriched in lipid metabolic and extracellular region.

## Introduction

With the number of all-age deaths closing to nine million in 2017, CAD is the leading cause of mortality worldwide (GBD 2017 Causes of Death Collaborators, 2018). Parallel to the high prevalence and vast number of deaths, CAD also places a crushing economic burden. According to the recent American Heart Association report, the medical costs and productivity losses of CVD are expected to grow from $555 billion in 2015 to $1.1 trillion in 2035 (Dunbar et al., 2018; Benjamin et al., 2019). Although CAD has become a major concern in global public health, there still a long way to fully understand the etiology of CAD before getting it under control.

In addition to lifestyle risk factors such as physical inactivity, unbalanced diet, smoking, alcohol, and obesity (Meier et al., 2019), genetic factors also play a pivotal role in CAD susceptibility (Musunuru and Kathiresan, 2019). Family and twin studies have demonstrated a strong genetic component of CAD, with heritability estimated between 40 and 60% (McPherson and Tybjaerg-Hansen, 2016; Khera and Kathiresan, 2017). To unravel the genetic underpinnings of CAD, multiple large-scale genetic studies have been performed. During the past decade, GWASs have identified more than 150 genetic loci at the commonly accepted genome-wide statistical significant threshold of *P* < 5 × 10^–8^ to account for multiple testing (Howson et al., 2017; Klarin et al., 2017; Nelson et al., 2017; van der Harst and Verweij, 2018). Considering the polygenic nature of CAD, more variants are expected to be identified in the near future owing to the rapid increase in sample size (Zhang et al., 2018). However, the success of GWAS has not been fully translated into an ability to find biological mechanisms and therapeutic targets behind these associations (Shu et al., 2018; Musunuru and Kathiresan, 2019).

There exist some difficulties in localizing the causal genes directly from the GWAS results. First, the lead variant identified by GWAS represents a set of variants in LD that usually spans large genomic regions (Farh et al., 2015). The complicated LD between SNPs and causative mutations is a major barrier to pinpoint the plausible causal genes. Second, the genes in the closest physical proximity to the top associated variants may be not the causal genes because of gene regulation (Smemo et al., 2014). The causal variants mediate the effect on disease risk through either a local effect on gene within the locus or action at a distance on a more remote gene. Therefore, the complexity of LD structure and distal regulation impedes our ability to identify causal genes from GWAS results. To address this issue, many GWAS-based computational methods aiming to prioritize the most likely causal genes have been developed (Rossin et al., 2011; He et al., 2013; Greene et al., 2015; Pers et al., 2015; Tasan et al., 2015; Gusev et al., 2016; Zhu et al., 2016; Shim et al., 2017). For example, *Sherlock*, *SMR*, and *TWAS* prioritize causal genes by combining GWAS and eQTL data; *DAPPLE* integrates GWAS data with PPI network to identify potential causal genes; *DEPICT* identifies causal genes through integrating GWAS and predicts gene functions; and *prix fixe*, *NetWAS*, and *GWAB* predict causal genes using co-function network, tissue-specific network, and human functional gene network, respectively.

**FIGURE 1:**
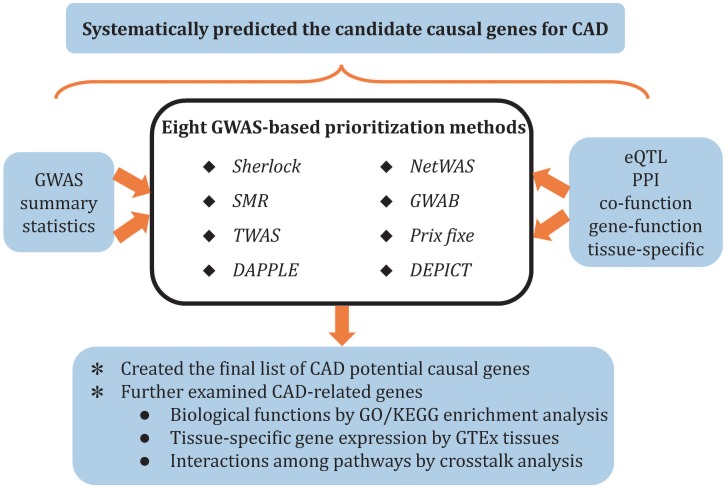
Overview of study pipelines.

In this study, we systematically prioritized the potential causal genes for CAD through eight cutting-edge methods (*Sherlock*, *SMR*, *DAPPLE*, *NetWAS*, *prix fixe*, *GWAB*, *DEPICT*, and *TWAS*), which are complementary with each other. Candidate causal genes were further characterized by pathway enrichment analysis, tissue-specific enrichment analysis, and pathway crosstalk analysis (Figure 1). This landscape of potential causal genes could provide information and evidence to elucidate the genetic mechanisms underlying CAD.

## Materials and Methods

### Genome-Wide Association Study Data of Coronary Artery Disease

We used summary statistics from a large-scale CAD GWAS in our study (Nelson et al., 2017). In brief, Nelson et al. (2017) performed a genome-wide association meta-analysis of the United Kingdom Biobank data with two published datasets (CARDIoGRAMplusC4D 1000 Genomes-imputed GWAS (Nikpay et al., 2015) and MIGen/CARDIoGRAM Exome chip study (Stitziel et al., 2016; Webb et al., 2017). The study comprised a total of 71,602 CAD cases and 260,875 controls and identified 304 independent variants, clustering 243 loci, associated with CAD at 5% FDR. More detailed information regarding sample recruitment, genotyping, quality control, and statistical analysis in provided in the original study (Nelson et al., 2017).

### Blood Expression Quantitative Trait Locus Data

We used eQTL summary data from the Consortium for the Architecture of Gene Expression (CAGE, *n* = 2,765 in peripheral blood) and the GTEx Consortium v7 release (*n* = 369 from whole blood). Briefly, Lloyd-Jones et al. (2017) performed this analysis of 2,765 samples from peripheral blood, with gene expression data observed from Illumina gene expression arrays (38,624 gene expression probes) and SNP genotype data imputed to the 1000 Genomes Phase 1 Version 3 reference panel (∼8 million SNPs). Information about tissue collection, genotyping, RNA quantification, and statistical analysis can be found in the original study of Lloyd et al. (2017). The GTEx project (GTEx Consortium, 2013) contained samples from 44 healthy tissues of 20- to 70-year-old human postmortem donors. For GTEx eQTL data (v7), whole blood tissues of 369 individuals were used, and gene expression levels measured by RNA-seq. SNP genotyping was performed using the Illumina OMNI SNP Arrays.

### Prioritization of Coronary Artery Disease Candidate Causal Genes

#### Sherlock Integrative Analysis

Based on the assumption that the expression change of a specific gene may contribute to CAD risk, we used *Sherlock* integrative analysis method to integrate SNP associations from CARDIoGRAMplusC4D consortium and blood eQTL from GTEx (He et al., 2013). *Sherlock* utilizes a Bayesian statistical framework to infer causal genes. It calculates the logarithm of Bayes factor (LBF) for each gene to represent the probability of association between specific gene and CAD. Bonferroni correction was used to correct the *P* value of genes identified by *Sherlock* integrative analysis. The corrected threshold of *P* value is 8.7 × 10^–6^ (there were 5,747 genes in the eQTL test).

#### Summary Data-Based Mendelian Randomization Analysis

Similar to *Sherlock*, *SMR* performs integrative analysis through integrating genetic associations from GWAS and eQTL data from CAGE (Zhu et al., 2016). Additionally, *SMR* uses *HEIDI* test to distinguish pleiotropy from linkage. Genes were considered as plausible causal gene only if they passed both *SMR* and *HEIDI* tests. The genome-wide significant threshold was defined as *P*_SMR_ < 5.24 × 10^–6^, because we retained 9,538 gene expression probes from the CAGE eQTL analysis.

#### Disease Association Protein–Protein Link Evaluator

Based on the “guilt by association” principle, many previous studies incorporated PPI network into GWAS analysis in order to prioritize candidate genes (Jia et al., 2012; Jia and Zhao, 2014), and *DAPPLE* is one of the popular approaches (Rossin et al., 2011). It requires a set of association loci as input and prioritizes genes according to connectivity in the PPI network. Genes with a corrected *P* value less than 0.05 will be considered as potential causal genes.

#### Network-Wide Association Study

Network-Wide Association Study is a machine learning method that combines gene-level association with the tissue-specific interaction network (Greene et al., 2015). The network was built using 14,000 publications and low-throughput tissue-specific expression data, which could describe the gene–gene functional interactions within a specific tissue. In this study, we used VEGAS2 (Mishra and Macgregor, 2015) to convert SNP-based summary statistics into gene-based *P* values. We prioritize the genes in a network built from liver tissue and set 0.05 as *P* value threshold, which implied that genes with *P* value below this cutoff were treated as positive in *NetWAS*.

#### Prix Fixe Analysis

The *prix fixe* strategy uses co-function networks to prioritize genes from multiple disease-associated loci (Tasan et al., 2015). It identifies genes by evaluating the significance of combinations of genes, with one gene from each GWAS candidate locus, in a gene network. In this study, the top 100 index SNPs from CAD GWAS were used as input for *prix fixe* (as *prix fixe* only accepts a maximum of 100 SNPs as input). By averaging the importance measurements, *prix fixe* (PF) score was obtained to prioritize candidate genes.

#### Genome-Wide Association Boosting

Holding the view that the genes associated with a disease tend to be functionally together, *GWAB* prioritizes candidate disease genes by integrating the GWAS data and human functional gene network (Shim et al., 2017). Unlike other network-based approach, *GWAB* has an advantage that it integrates complementary information from both population-based approach and molecular profiling approach to identify disease-associated genes. Therefore, it can make the best of information identified from functional experiments apart from the statistical association from GWAS. Shim et al. (2017) used GWAS data from Schunkert et al. (2011) as input and referenced genes from three disease gene databases (OMIM, DO, and CADgeneDB) as benchmark to predict for disease-associated genes. We therefore included Jung Eun Shim’s results into our study.

#### Transcriptome-Wide Association Studies

The *TWAS* (Gusev et al., 2016) is a powerful strategy that integrates SNP–expression correlation, GWAS summary statistics, and LD reference panels to identify genes whose *cis*-regulated expression is associated with disease risk. This method can be conceptualized as having imputed expression data for all cases and controls who participated in GWAS. It then correlated the imputed gene expression with traits to perform a TWAS and identified significant expression-trait associations. Mancuso et al. (2017) have integrated gene expression measurements from 45 expression panels with 30 large-scale summary GWAS data to gain insight into the role of expression in the etiology of complex traits by using *TWAS*. We therefore included Mancuso’s results into our study.

#### Data-Driven Expression Prioritized Integration for Complex Traits

The *DEPICT* tool predicts the most likely causal genes by integrating GWAS and data and gene functions (Pers et al., 2015). Predefined gene sets and gene-transcriptional component matrix developed from gene expression data are used to obtain gene function. van der Harst and Verweij (2018) have prioritized candidate causal genes for CAD using *DEPICT*. They defined sentinel SNPs as the most significant variant in a 1-Mb region that was independent from other sentinel SNPs (*r*^2^ < 0.1), which was consistent with Pers et al. (2015). Therefore, we included Van der Harst’s results (van der Harst and Verweij, 2018) (prioritized causal gene list) into our study.

### Ranking the Potential Causal Genes

We used eight different approaches to predict the potential causal genes of CAD. As they are complementary with each other, we assigned equal weights to all methods. Each candidate gene will receive a score of 1 if it is identified by any of the above-mentioned approaches. A cumulative scoring strategy was performed to rank the causal genes. For example, if a gene was only identified by *Sherlock* analysis, the total score of this gene was 1 point. If a gene was identified by both *Sherlock* and *SMR*, the total score of this gene was 2 points. The higher the score of a gene, the more consistent its evidence for identification across the methods and thus the greater the likelihood of involvement in disease susceptibility.

### Gene Ontology and Kyoto Encyclopedia of Genes and Genomes Enrichment Analysis

We performed GO analysis to test if the identified potential causal genes were significantly enriched in specific functional categories by using DAVID (Huang da et al., 2009a,b). Three GO terms, including biological process (BP), cellular component (CC), and molecular function (MF) were used. We also conducted KEGG enrichment analysis using ToppGene (Chen et al., 2009). KEGG pathways and GO terms with adjusted *P* values less than 0.05 [Benjamini and Hochberg (BH) method for correction of multiple testing] were considered as statistical significant.

### Tissue-Specific Enrichment Analysis

In order to identify the most relevant tissue for CAD candidate causal genes, we calculated a *pSI* score for each gene to represent its enrichment in a specific tissue. Details of *pSI* score calculation can refer to the original publication (Wells et al., 2015). For each tissue, genes with *pSI* < 0.05 are considered as significantly enriched in the tissue. The overlap between CAD potential causal genes and the genes enriched in each tissue was estimated by Fisher’s exact test. Bonferroni correction was used to adjust for multiple testing. The significant threshold was defined as *P* < 0.002, because 25 tissues were tested.

### Analysis of Protein–Protein Interaction and Co-Expression Network

To explore the physical interaction among the proteins encoded by the potential causal genes, we used human PPI data from STRING (Szklarczyk et al., 2017). Additionally, we further investigated the co-expressed genes of the prioritized causal genes using GeneMANIA (Montojo et al., 2014). Cytoscape was employed to visualize the network (Shannon et al., 2003). We assumed that the gene association density of CAD potential causal gene set was higher than that of random gene sets. To test this hypothesis, we generated 1,000 random gene sets, each with the same number of genes as CAD potential causal gene set, and we assessed the significance using *Z*-test.

### Pathway Crosstalk Analysis

In order to explore the interactions among significantly enriched pathways, we further performed pathway crosstalk analysis. Two pathways were considered to crosstalk if they share a proportion of genes prioritized in our study. We employed two measurements to indicate the overlap of a pair of pathways: the Jaccard coefficient $\left(\text{JC};\mathrm{JC}=\frac{\left|A\cap B\right|}{\left|A\cup B\right|}\right)$ and the overlap coefficient $\left(\text{OC};\mathrm{OC}=\frac{\left|A\cap B\right|}{\min\left(\left|A\right|,\left|B\right|\right)}\right)$, where *A* and *B* represent the number of candidate genes in the two pathways. In order to preclude non-specific inclusion of crosstalk, we set up the following rules: (1) only pathways with adjusted *P* values < 0.05 were included; (2) only pathways contained at least three candidate genes were included; and (3) pathway pairs with less than two overlapped candidate genes were removed. We visualized the results of pathway crosstalk analysis using Cytoscape (Shannon et al., 2003).

## Results

### Potential Causal Genes Identified by *Sherlock*

To identify genes whose expression level change may confer risk of CAD, we systematically integrated genetic associations from the largest GWAS of CAD so far with blood eQTL data from GTEx using *Sherlock*, a Bayesian statistical framework. We identified 17 potential causal genes whose expression level may affect CAD risk at *P* < 8.7 × 10^–6^ (Bonferroni corrected *P* < 0.05, Supplementary Table S1). For each gene, at least one SNP showed significant association with the expression of this gene and CAD risk simultaneously, implying that these SNPs may exert their effects through regulating gene expression.

### Potential Causal Genes Identified by *SMR*

We further utilized a different integrative analysis method, *SMR*, to identify CAD risk genes through integrating CAD GWAS and blood eQTL data from CAGE. *SMR* identified six genes at *P* < 5.24 × 10^–6^. However, *VAMP8* and *MAT2A* did not pass *HEIDI* test (*P* < 0.05), and only four genes (*PSRC1*, *LIPA*, *SWAP70*, and *NT5C2*) were retained (Supplementary Table S2). Intriguingly, three of them (*PSRC1*, *LIPA*, and *NT5C2*) were also identified by *Sherlock*, bringing further evidence to support that they might be the authentic risk genes for CAD.

### Potential Causal Genes Identified by *DAPPLE*

Disease Association Protein–Protein Link Evaluator prioritizes disease-associated genes through using PPI data. A total of 58 genes were identified at corrected *P* < 0.05 (Supplementary Table S3). The top prioritized genes included *SVEP1*, *APOB*, *FES*, *SLC22A4*, *PLG*, *ABCG5*, *FLT1*, *HNF1A*, *LDLR*, and *UBE2Z*. GO analysis showed that these prioritized genes were enriched in lipid metabolism, transportation, and storage-related pathways (Supplementary Table S4).

### Potential Causal Genes Identified by *NetWAS*

We further mapped the gene-wide results to liver-specific network and classified by machine learning models as implemented by *NetWAS*. The top 50 genes were included in our further analysis (Supplementary Table S5). The most significant GO terms enriched among promising candidates genes were “protein binding” (corrected *P* = 7.6 × 10^–8^), “cytosol” (corrected *P* = 2.0 × 10^–7^), and “nucleoplasm” (corrected *P* = 4.3 × 10^–7^) (Supplementary Table S6).

### Potential Causal Genes Identified by *Prix Fixe*

We further predicted CAD causal genes using *prix fixe*, which incorporates the functionally coherent subnetworks into network analysis. Ten genes were identified by *prix fixe*, including *PCSK9*, *DHCR24*, *SOD2*, *LPA*, *PLG*, *MAP3K4*, *CDKN2B*, *CDKN2A*, *ADORA2A*, and *DERL3* (Supplementary Table S7). Of note, *PCSK9* ranked the highest score among those genes. Genetic evidence indicated that individuals with inactivating mutations in *PCSK9* have decreased levels of circulating *LDL* and CAD risk, which led to the two monoclonal antibodies that inhibit *PCSK9* to be approved by the United States Food and Drug Administration (Cohen et al., 2006). The finding of *PCSK9* further reinforced other genes identified by *prix fixe* as promising causal genes for CAD.

### The Integrated Landscape of Causal Genes in Coronary Artery Disease

We utilized different approaches (including *Sherlock*, *SMR*, *DAPPLE*, *NetWAS*, *prix fixe*, *GWAB*, *DEPICT*, and *TWAS*) to prioritize the potential causal genes for CAD. In order to obtain the global landscape of plausible causal genes, we also integrated candidate causal genes identified by previous studies. Studies included in our further analysis are summarized as follows: (1) Causal genes predicted by Pavlides et al. using SMR (Pavlides et al., 2016). By integrating Westra blood eQTL data (Westra et al., 2013) and CARDIoGRAMplusC4D 1000 Genomes-based GWAS data (60,801 cases and 123,504 controls) (Nikpay et al., 2015), Pavlides et al. (2016) used SMR to prioritize genes and identify five candidate genes (*VAMP8*, *SWAP70*, *IL6R*, *ATP5G1*, and *EIF2B2*) that passed both *SMR* and *HEIDI* test (Supplementary Table S8). (2) Causal genes predicted by *GWAB* (Shim et al., 2017). Jung Eun Shim et al. used GWAS data from CARDIoGRAMplusC4D consortium (22,233 cases and 64,762 controls) as input and references genes from three disease gene databases (OMIM, DO, and CADgeneDB) as benchmark to predict for disease-associated genes. After network boosting, 35 genes from the largest component of the network were significantly associated with CAD (GWAB score > 7.3) (Supplementary Table S9). (3) Causal genes predicted by *DEPICT* (van der Harst and Verweij, 2018). Gusev et al. (2016) predicted the causal genes for CAD recently. A total of 433 genes were identified as plausible candidate genes for CAD (FDR < 0.01) (Supplementary Table S10). (4) Causal genes predicted by *TWAS*. Mancuso et al. (2017) integrated CARDIoGRAM consortium GWAS data (Schunkert et al., 2011) with various expression panels and identified 12 genes significantly associated with CAD (Supplementary Table S11).

**FIGURE 2:**
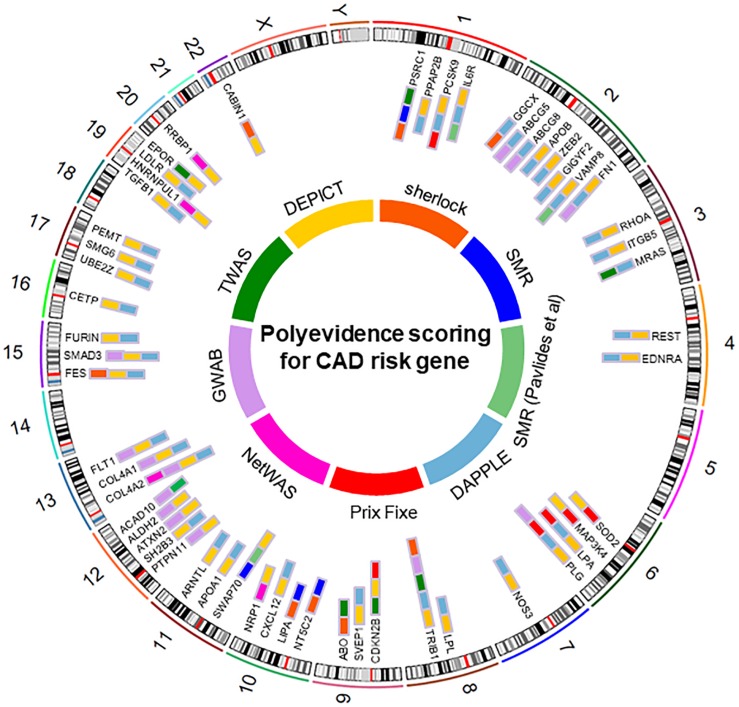
Top causal genes identified in this study.

Given that the results of the eight analytical methods are correlated but not identical, genes genuinely involved in disease susceptibility would be expected to show consistent results across several methods. We therefore adopted the method of cumulative scoring strategy and ranked the plausible causal genes by their frequency of occurrences in the results of different approaches. We summarized the results in Table 1 and generated the landscape of potential causal genes in CAD (Figure 2). This yielded 55 candidate genes with total score ≥2, among which 15 genes were captured by at least three analysis methods. Therefore, these 15 genes (*LPL*, *COL4A2*, *PLG*, *CDKN2B*, *COL4A1*, *FES*, *FLT1*, *FN1*, *IL6R*, *LPA*, *PCSK9*, *PSRC1*, *SMAD3*, *SWAP70*, and *VAMP8*) were considered as the most promising causal genes for CAD. We queried the NHGRI-EBI GWAS Catalog (Buniello et al., 2019) about the candidate genes to assess their potential pleiotropic effects. Notably, many of these genes provided evidence of pleiotropic effect of lipid traits, which confirmed the significant role of lipid metabolism in CAD.

**TABLE 1:** Summary of 55 prioritized causal genes.

No.	Gene	Locus	First identified by GWAS	Potential biological function	Other related traits*	Methods
1	*LPL*	8p21.3	2017–2018	Triglyceride-rich lipoproteins	HDL, TG	Sherlock, GWAB, TWAS, DEPICT, DAPPLE
2	*COL4A2*	13q34	2011	Cellular proliferation and vascular remodeling	Colorectal cancer, coronary artery calcification, DBP, TC	NetWAS, GWAB, DEPICT, DAPPLE
3	*PLG*	6q26	2015	Cellular proliferation and vascular remodeling	Blood protein measurement	GWAB, DEPICT, DAPPLE, prix fixe
4	*CDKN2B*	9p21.3	2007–2008	Cellular proliferation and vascular remodeling	Cancer, heart failure, diabetes mellitus, stroke, atrial fibrillation, mortality	TWAS, DEPICT, prix fixe
5	*COL4A1*	13q34	2011	Cellular proliferation and vascular remodeling	Arterial stiffness measurement, stroke	GWAB, DEPICT, DAPPLE
6	*FES*	15q26.1	2017–2018	Uncertain	SBP, DBP	Sherlock, DEPICT, DAPPLE
7	*FLT1*	13q12.3	2017–2018	Cellular proliferation and vascular remodeling	–	GWAB, DEPICT, DAPPLE
8	*FN1*	2q35	2017–2018	Coagulation	Blood protein, SBP, DBP	GWAB, DEPICT, DAPPLE
9	*IL6R*	1q21.3	2015	Inflammation	C-reactive protein, rheumatoid arthritis	SMR (Pavlides), DEPICT, DAPPLE
10	*LPA*	6q26	2011	LDL cholesterol and lipoprotein (a)	Lipoprotein a, LDL, colorectal cancer	DEPICT, DAPPLE, prix fixe
11	*PCSK9*	1p32.3	2015	LDL cholesterol and lipoprotein (a)	LDL, TC	DEPICT, DAPPLE, prix fixe
12	*PSRC1*	1p13.3	2007–2008	LDL cholesterol and lipoprotein (a)	LDL, TC, HDL	Sherlock, SMR, TWAS
13	*SMAD3*	15q22.33	2015	Cellular proliferation and vascular remodeling	Asthma, Crohn’s disease, inflammatory bowel disease	GWAB, DEPICT, DAPPLE
14	*SWAP70*	11p15.4	2015	Cellular proliferation and vascular remodeling	SBP, DBP	SMR, SMR (Pavlides), DEPICT
15	*VAMP8*	2p11.2	2015	Cellular proliferation and vascular remodeling	Prostate cancer	SMR (Pavlides), DEPICT, DAPPLE
16	*ABCG5*	2p21	2017–2018	LDL cholesterol and lipoprotein (a)	TC, LDL	GWAB, DAPPLE
17	*ABCG8*	2p21	2017–2018	LDL cholesterol and lipoprotein (a)	TC, LDL	GWAB, DAPPLE
18	*ABO*	9q34.2	2011	Uncertain	TC, LDL, von Willebrand factor, hematocrit, hemoglobin, factor VIII, alkaline phosphatase, coagulation factor	Sherlock, TWAS
19	*ACAD10*	12q24.12	2013–2014	Uncertain	Esophageal cancer	GWAB, TWAS
20	*ALDH2*	12q24.12	2013–2014	Uncertain	Ischemic stroke, alcohol consumption, uric acid	GWAB, DEPICT
21	*APOA1*	11q23.3	2011	Triglyceride-rich lipoproteins	HDL	DEPICT, DAPPLE
22	*APOB*	2p24.1	2017–2018	LDL cholesterol and lipoprotein (a)	TC, LDL	DEPICT, DAPPLE
23	*ARNTL*	11p15.3	2017–2018	Uncertain	C-reactive protein, TC	DEPICT, DAPPLE
24	*ATXN2*	12q24.12	2013–2014	Uncertain	PAD	GWAB, DEPICT
25	*CABIN1*	22q11.23	–	–	–	Sherlock, DEPICT
26	*CETP*	16q13	2017–2018	LDL cholesterol and lipoprotein (a)	HDL, LDL, TC, TG, alcohol drinking. BMI	DEPICT, DAPPLE
27	*CXCL12*	10q11.21	2011	Inflammation	White blood cell count, eosinophil count, erythrocyte count, schizophrenia	DEPICT, DAPPLE
28	*EDNRA*	4q31.22	2015	Vascular tone and nitric oxide signaling	Large artery stroke, pancreatic carcinoma, PAD, SBP, PP	DEPICT, DAPPLE
29	*EPOR*	19p13.2	–	–	Erythropoietin	TWAS, DEPICT
30	*FURIN*	15q26.1	2017–2018	Uncertain	SBP, DBP, ischemic stroke, schizophrenia	DEPICT, DAPPLE
31	*GGCX*	2p11.2	2015	Cellular proliferation and vascular remodeling	Eosinophil count	Sherlock, DAPPLE
32	*GIGYF2*	2q37.1	2017–2018	Uncertain	Schizophrenia	DEPICT, DAPPLE
33	*HNRNPUL1*	19q13.2	–	–	Heel bone mineral density	NetWAS, DEPICT
34	*ITGB5*	3q21.2	2017–2018	Uncertain	SBP	DEPICT, DAPPLE
35	*LDLR*	19p13.2	2011	LDL cholesterol and lipoprotein (a)	LDL, TC, abdominal aortic aneurysm	DEPICT, DAPPLE
36	*LIPA*	10q23.31	2011	LDL cholesterol and lipoprotein (a)	–	Sherlock, SMR
37	*MAP3K4*	6q26	–	–	Blood protein	DEPICT, prix fixe
38	*MRAS*	3q22.3	2009–2010	Inflammation	BMI, SBP	TWAS, DAPPLE
39	*NOS3*	7q36.1	2015	Vascular tone and nitric oxide signaling	SBP, DBP, stroke	DEPICT, DAPPLE
40	*NRP1*	10p11.22	2017–2018	Uncertain	Migraine	NetWAS, DEPICT
41	*NT5C2*	10q24.33	2011	Uncertain	SBP, DBP, PP, BMI, schizophrenia, smoking behavior	Sherlock, SMR
42	*PEMT*	17p11.2	2011	Uncertain	BMI, WHR	DEPICT, DAPPLE
43	*PPAP2B*	1p32.2	2011	LDL cholesterol and lipoprotein (a)	Reticulocyte count, heel bone mineral density	DEPICT, DAPPLE
44	*PTPN11*	12q24.13	2013–2014	Uncertain	Eosinophil count, SBP, DBP, platelet count, reticulocyte count, smoking	GWAB, DEPICT
45	*REST*	4q12	2017–2018	Uncertain	Risk-taking behavior	DEPICT, DAPPLE
46	*RHOA*	3p21.31	2017–2018	Uncertain	Cognitive function, self-reported educational attainment, intelligence	DEPICT, DAPPLE
47	*RRBP1*	20p12.1	2017–2018	Uncertain	Migraine, height	NetWAS, DEPICT
48	*SH2B3*	12q24.12	2015	Inflammation	SBP, DBP, BMI, LDL, HDL, alcohol consumption, autoimmune disease, colorectal cancer, endometrial carcinoma, eosinophil count, eosinophil count, fibrinogen, hemoglobin, Ischemic stroke, leukocyte, lymphocyte, platelet, rheumatoid arthritis, type I diabetes mellitus	DEPICT, DAPPLE
49	*SMG6*	17p13.3	2011	Uncertain	BMI, bone density, schizophrenia, platelet, smoking	DEPICT, DAPPLE
50	*SOD2*	6q25.3	–	–	Self-reported educational attainment, age at menopause	DEPICT, prix fixe
51	*SVEP1*	9q31.3	2017–2018	Uncertain	Bipolar disorder, SBP	DEPICT, DAPPLE
52	*TGFB1*	19q13.2	2017–2018	Uncertain	T2D	DEPICT, DAPPLE
53	*TRIB1*	8q24.13	2017–2018	Triglyceride-rich lipoproteins	HDL, LDL, TG, glomerular filtration rate	DEPICT, DAPPLE
54	*UBE2Z*	17q21.32	2011	Uncertain	Educational attainment, T2D	DEPICT, DAPPLE
55	*ZEB2*	2q22.3	2015	Uncertain	Renal cell carcinoma, schizophrenia, self-reported educational attainment	DEPICT, DAPPLE

*The association with other traits or diseases was identified from the National Human Genome Research Institute-European Bioinformatics Institute GWAS catalog. LDL, low-density lipoprotein; HDL, high-density lipoprotein; TG, triglyceride; DBP, diastolic blood pressure; SBP, systolic blood pressure; TC, total cholesterol; PAD, peripheral arterial disease; PP, pulse pressure; BMI, body mass index; WHR, waist–hip ratio; T2D, type 2 diabetes.

**FIGURE 3:**
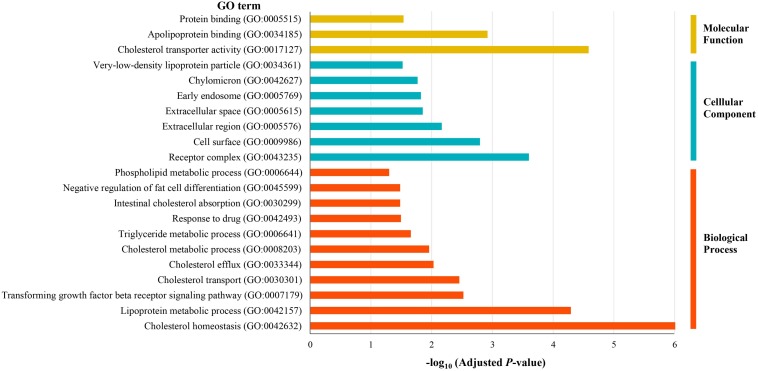
Gene Ontology enrichment analysis of the prioritized causal genes of CAD.

To identify the biological roles of the 55 potential causal genes, we performed GO enrichment analysis using DAVID. We found that candidate genes were significantly enriched in the GO term such as cholesterol homeostasis (corrected *P* < 9.6 × 10^–7^), lipoprotein metabolic process (corrected *P* < 5.1 × 10^–5^), receptor complex (corrected *P* < 2.5 × 10^–4^), cholesterol transporter activity (corrected *P* < 2.6 × 10^–5^), extracellular region (corrected *P* < 6.8 × 10^–3^), and apolipoprotein binding (corrected *P* < 1.2 × 10^–3^) (Figure 3). These results indicated that lipid metabolic and extracellular region might play a critical role in the pathophysiology of CAD.

**FIGURE 4:**
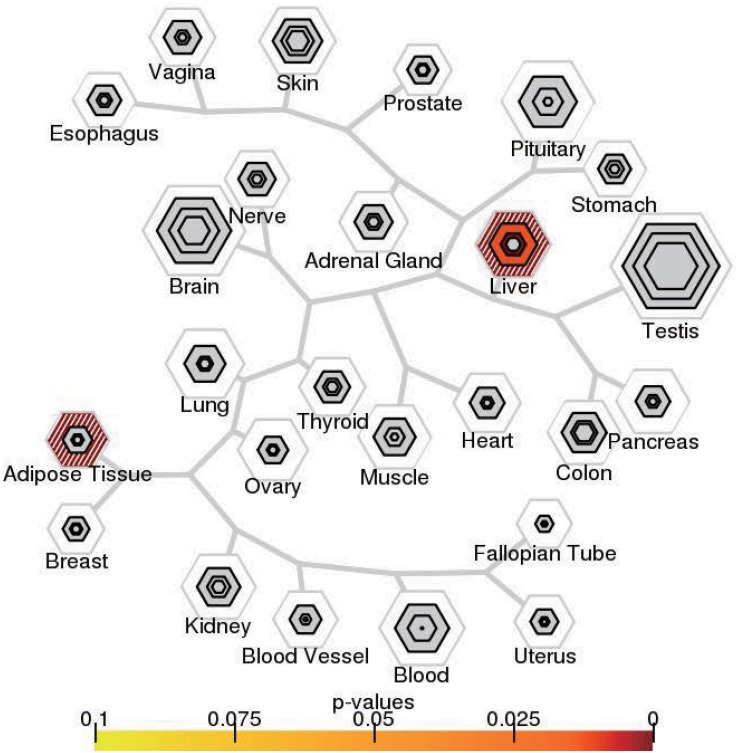
Tissue-specific expression enrichment of CAD potential causal genes. The 53 tissues in GTEx were grouped into 25 broad tissue types. The size of the hexagon was scaled to the number of genes meeting the *pSI* threshold, and its color indicates results of Fisher’s exact test.

**FIGURE 5:**
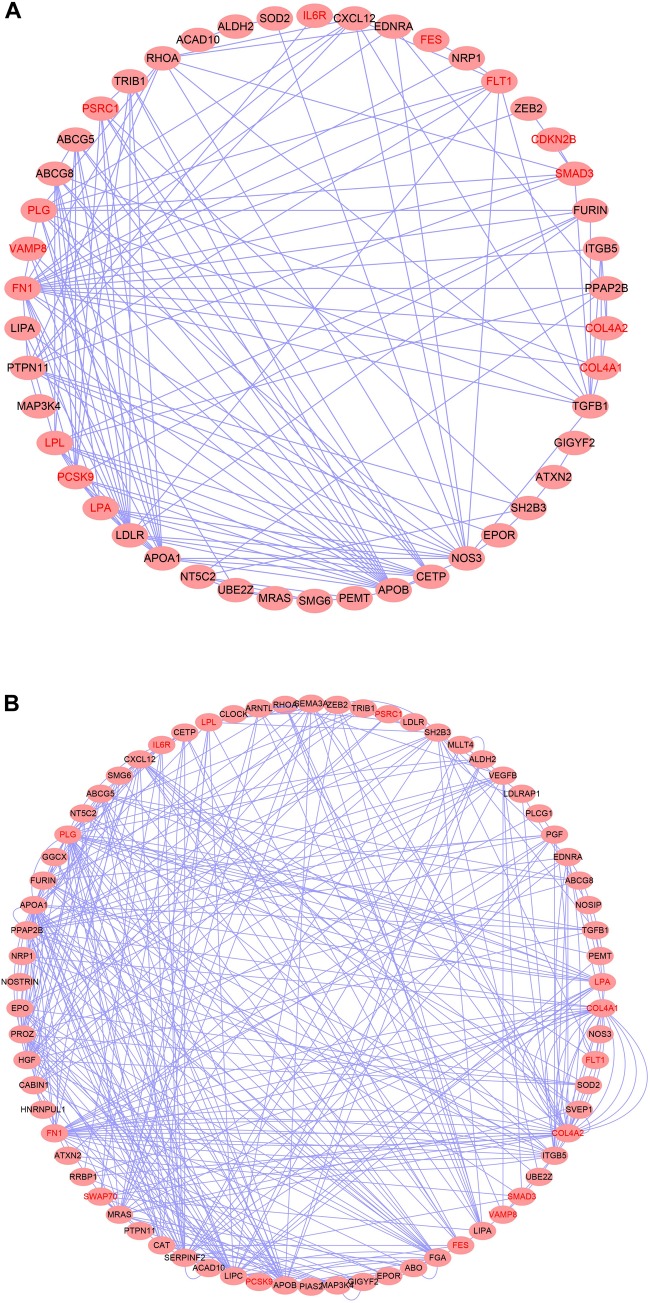
Top causal genes encoded a densely interconnected PPI **(A)** and co-expression **(B)** network.

We then examined the most relevant tissue of CAD potential causal genes using tissue-specific enrichment analysis. As shown in Figure 4, these genes tended to be significantly overexpressed in liver (*P* = 2.2 × 10^–6^) and adipose tissue (*P* = 2.8 × 10^–7^). Considering genes are usually act synergistically to exert their biological function, also called as “guilt by association” principle, we further performed network analysis using PPI and expression data. As shown in Figure 5, genes in the PPI network (Figure 5A) and co-expression network (Figure 5B) demonstrated more interaction among themselves than what would be expected for a random set of genes of the same size (*P* < 1.0 × 10^–16^). Dysregulation of any gene in this highly interconnected network will affect the function of the network, which eventually lead to pathogenesis of CAD.

### Kyoto Encyclopedia of Genes and Genomes Enrichment Analysis and Pathway Crosstalk Analysis

Identifying KEGG pathways enriched in the candidate genes may provide further insight of the molecular mechanisms related to CAD. Table 2 shows the 24 significant enriched pathways. Among these, the top five pathways are fat digestion and absorption (*P*_BH_ = 6.63 × 10^–5^), AGE-RAGE signaling pathway in diabetic complications (*P*_BH_ = 1.92 × 10^–4^), pathways in cancer (*P*_BH_ = 1.12 × 10^–3^), PI3K-Akt signaling pathway (*P*_BH_ = 1.90 × 10^–3^), and focal adhesion (*P*_BH_ = 3.96 × 10^–3^).

**TABLE 2:** KEGG enrichment analysis in CAD prioritized causal genes.

Pathway	ID	*P* value	*P*_BH_ value	Candidate genes in the pathway
Fat digestion and absorption	hsa04975	5.72 × 10^–7^	6.63 × 10^–5^	*PLPP3*, *APOA1*, *APOB*, *ABCG5*, *ABCG8*
AGE-RAGE signaling pathway in diabetic complications	hsa04933	2.60 × 10^–6^	1.92 × 10^–4^	*TGFB1*, *COL4A1*, *COL4A2*, *FN1*, *NOS3*, *SMAD3*
Pathways in cancer	hsa05200	2.35 × 10^–5^	1.12 × 10^–3^	*TGFB1*, *COL4A1*, *RHOA*, *COL4A2*, *CDKN2B*, *FN1*, *CXCL12*, *EDNRA*, *SMAD3*
PI3K-Akt signaling pathway	hsa04151	5.86 × 10^–5^	1.90 × 10^–3^	*COL4A1*, *COL4A2*, *EPOR*, *FLT1*, *FN1*, *ITGB5*, *NOS3*, *IL6R*
Focal adhesion	hsa04510	1.37 × 10^–4^	3.96 × 10^–3^	*COL4A1*, *RHOA*, *COL4A2*, *FLT1*, *FN1*, *ITGB5*
Proteoglycans in cancer	hsa05205	1.53 × 10^–4^	4.10 × 10^–3^	*TGFB1*, *RHOA*, *PTPN11*, *MRAS*, *FN1*, *ITGB5*
ECM-receptor interaction	hsa04512	3.20 × 10^–4^	6.30 × 10^–3^	*COL4A1*, *COL4A2*, *FN1*, *ITGB5*
Small cell lung cancer	hsa05222	3.51 × 10^–4^	6.32 × 10^–3^	*COL4A1*, *COL4A2*, *CDKN2B*, *FN1*
TGF-beta signaling pathway	hsa04350	3.51 × 10^–4^	6.32 × 10^–3^	*TGFB1*, *RHOA*, *CDKN2B*, *SMAD3*
Amebiasis	hsa05146	5.83 × 10^–4^	9.45 × 10^–3^	*TGFB1*, *COL4A1*, *COL4A2*, *FN1*
Axon guidance	hsa04360	6.58 × 10^–4^	1.03 × 10^–2^	*RHOA*, *PTPN11*, *FES*, *CXCL12*, *NRP1*
Glycerolipid metabolism	hsa00561	1.69 × 10^–3^	1.99 × 10^–2^	*PLPP3*, *LPL*, *ALDH2*
Colorectal cancer	hsa05210	1.78 × 10^–3^	2.06 × 10^–2^	*TGFB1*, *RHOA*, *SMAD3*
FoxO signaling pathway	hsa04068	1.91 × 10^–3^	2.06 × 10^–2^	*TGFB1*, *CDKN2B*, *SMAD3*, *SOD2*
Phospholipase D signaling pathway	hsa04072	2.75 × 10^–3^	2.45 × 10^–2^	*RHOA*, *PTPN11*, *MRAS*, *PLPP3*
Bile secretion	hsa04976	2.88 × 10^–3^	2.48 × 10^–2^	*LDLR*, *ABCG5*, *ABCG8*
Chronic myeloid leukemia	hsa05220	2.88 × 10^–3^	2.48 × 10^–2^	*TGFB1*, *PTPN11*, *SMAD3*
HTLV-I infection	hsa05166	3.52 × 10^–3^	2.80 × 10^–2^	*TGFB1*, *CDKN2B*, *MRAS*, *SMAD3*, *NRP1*
Endocytosis	hsa04144	3.76 × 10^–3^	2.85 × 10^–2^	*TGFB1*, *RHOA*, *FLT1*, *LDLR*, *SMAD3*
phosphatidylcholine (PC) biosynthesis, PE → PC	hsa00091	4.03 × 10^–3^	2.92 × 10^–2^	*PEMT*
Vitamin digestion and absorption	hsa04977	4.12 × 10^–3^	2.93 × 10^–2^	*APOA1*, *APOB*
Cytokine–cytokine receptor interaction	hsa04060	4.42 × 10^–3^	3.11 × 10^–2^	*TGFB1*, *EPOR*, *FLT1*, *IL6R*, *CXCL12*
Rheumatoid arthritis	hsa05323	5.60 × 10^–3^	3.69 × 10^–2^	*TGFB1*, *FLT1*, *CXCL12*
HIF-1 signaling pathway	hsa04066	7.71 × 10^–3^	4.77 × 10^–2^	*FLT1*, *NOS3*, *IL6R*

**FIGURE 6:**
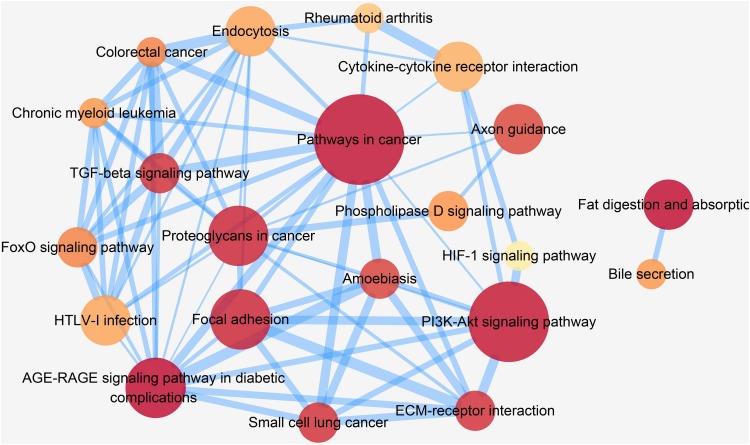
Crosstalk network among CAD prioritized causal genes enriched pathways. In this figure, each node represents a pathway, and each edge represents crosstalk between pathways. The color of each node is proportional to the adjusted *P* [Benjamini and Hochberg (BH) method] value. Darker color represents lower *P*_BH_ value. The size of each node is proportional to the number of CAD prioritized causal genes in the corresponding pathway. The width of each edge is proportional to the mean value of the two coefficients (JC and OC). Larger edge width represents higher proportion of overlap between pathways.

In order to gain deep understanding of how these pathways are related, we further performed a pathway crosstalk analysis. There were 22 pathways containing at least three candidate genes and met the inclusion criteria for crosstalk analysis. A total of 73 edges connected between any two of these pathways, and these edges represented the overlapping level, which was measured according to the average score of JC and OC. As shown in Figure 6, the largest crosstalk module comprised 20 pathways. By considering the topological characteristics, we identified five key pathways (pathways in cancer, AGE-RAGE signaling pathway in diabetic complications, proteoglycans in cancer, endocytosis, and PI3K-Akt signaling pathway), which had the highest betweenness centrality and degree value (Supplementary Table S12). Previous studies had provided some clues about their role in pathogenesis of CAD (Chen et al., 2016; Kay et al., 2016; Koene et al., 2016).

## Discussion

Although 163 loci have now been associated with CAD at a genome-wide level of significance (Erdmann et al., 2018), the reported loci usually span a large chromosomal region and contain many genes. Thus, it is difficult to pinpoint the causal gene. Besides, owing to the complexity of gene regulatory and LD pattern, this problem becomes even more complicated. Consequently, incorporating other source of prior knowledge is necessary to narrow down the CAD candidate genes. In this study, we conducted a comprehensive integrative analysis and prioritized the potential causal genes for CAD using the latest GWAS and other omics data from different layers. A total of 55 plausible causal genes were identified, among which 15 genes ranked the highest priority because of their consistent evidence from different data-driven approaches. Many of these genes were involved in in lipid metabolic and extracellular related BP. Moreover, they were enriched in liver and adipose tissue. Further, KEGG and crosstalk analysis also revealed several key pathways participating in the pathogenesis of CAD.

Our comprehensive analysis predicted 55 CAD causal genes. Many of these genes are well-known CAD driver genes and participant in lipid metabolic process (*LPL*, *LPA*, *PCSK9*, *APOA1*, *APOB*, *CETP*, *LDLR*, and *LIPA*). Some of them have been translated into the targets of commercialized drugs (*APOB* and *PCSK9*). Besides, GO enrichment analysis also highlight the importance of lipid involved in CAD pathogenesis. However, targeting novel pathways instead of established CAD risk factors, such as lipid levels, may facilitate the drug development and catalyze novel CAD therapeutic options. Our GO analysis also pinpointed that the identified potential causal genes were enriched in extracellular region apart from lipid metabolic. Extracellular matrix has a critical role in cell adhesion, integrity, and communication. Changes in the extracellular space have been implicated in the pathogenesis of atherosclerosis and restenosis (Chistiakov et al., 2013). Previous network analysis also revealed the key role of extracellular region involved in CAD development and the relationship between other molecular mechanisms, such as inflammatory response and complement and coagulation (Zhao et al., 2016).

In addition to identifying the well-known CAD driver genes, our comprehensive analysis also detected novel or less well-studied plausible causal genes for CAD. For example, *MAP3K4* encodes Mitogen-Activated Protein Kinase 4, which is the central core of each mitogen-activated protein (MAP) kinase pathway. Four well-characterized MAP kinase pathways, including ERK1/2, JNK, p38, and ERK5, have been reported their role in different aspects of cardiac regulation, from development to pathological remodeling (Wang, 2007). In line with our study, Ballouz et al. (2014) analyzed CARDIoGRAMplusC4D GWAS data by *Gentrepid* and found that *MAP3K4* was one of the possible causal genes of CAD. Another candidate, *CABIN1*, plays a pivotal role in the T cell receptor-mediated signal transduction pathway and inhibits calcineurin-mediated signal transduction. Calcineurin lies at the intersection of protein phosphorylation and calcium signaling cascades and contributes to pathological hypertrophic remodeling (Letavernier et al., 2012; Parra and Rothermel, 2017). Another identified gene, *EPOR*, encodes erythropoietin (EPO) receptor. EPO has shown its effect beyond hematopoiesis, such as suppression of atherosclerosis (Ueba et al., 2013) and prevention of cardiac apoptosis (Nakamura et al., 2009). In addition, although EpoR agonists drugs are initially aimed at treating anemia, it is starting to demonstrate the sign of pleiotropic effects for treating a wide range of complex disorders, including CVD, neurodegenerative disorders, spinal cord injury, and diabetic retinopathy (Sanchis-Gomar et al., 2013). *HNRNPUL1* encodes a nuclear RNA-binding protein and involved in mRNA splicing pathway. LeBlanc et al. (2016) integrated GWAS data of CAD and several CVD risk factors and used a shared polygenic signal-informed statistical framework to discover novel CAD genes. Totally, they identified 67 novel loci associated with CAD. Among these novel loci, rs12459996 near *CYP2F1* also showed eQTL effect on *HNRNPUL1* gene expression in blood tissue (*P* = 3.86 × 10^–16^), which indicated that this locus may exert its effect on CAD susceptibility through regulation of *HNRNPUL1* expression. Superoxide Dismutase 2 (*SOD2*) is a well-known marker of endothelial dysfunction. Several candidate gene studies have revealed that mutations or polymorphisms of *SOD2* gene are associated with CAD risk (Mollsten et al., 2009; Tian et al., 2012). Moreover, studies on Sod2-deficient mice demonstrated accelerated atherosclerosis (Zhou et al., 2012) and atherosclerotic plaque instability (Vendrov et al., 2017). These lines of evidence suggested that *SOD2* might act an important role in CAD pathogenesis by regulating atherosclerosis and endothelial function.

Pathway enrichment analysis of the prioritized genes further confirmed some results of previous studies. Some key drivers of CAD identified by previous network analysis were cancer-related genes (Zhao et al., 2016). Although recognized as two separate diseases, CAD and cancer possess various similarities, from common risk factors to shared BP (Koene et al., 2016). The oxidative stress and chronic inflammation underlie both of the diseases. In addition, a growing number of evidence support a role for statins, angiotensin-converting enzyme inhibitor/angiotensin receptor blockers (ACEIs/ARBs), and aspirin in cancer prevention (Masoudkabir et al., 2017). Anti-CD47 antibody treatment may become a novel therapeutic strategy for CAD by promoting efferocytosis (Kojima et al., 2016). This convergent evidence indicated that pathways of cancer might take part in the pathogenesis of CAD. AGE-RAGE signaling pathway actively participates in inflammation and immune response. Miao et al. (2019) identified 413 differential expressed genes from two gene expression datasets of CAD cases and healthy controls, and they highlighted the role of AGE-RAGE signaling pathway in CAD pathogenesis. Extensive evidence is building to implicate AGE-RAGE in the pathogenesis of vascular perturbation, which stimulate processes that lead to the development of arterial stiffness (Senatus and Schmidt, 2017). AGE/RAGE signaling also demonstrated its role in diabetes-mediated vascular calcification (Kay et al., 2016). Calcium deposits in coronary arteries may weaken vasomotor responses and alter atherosclerotic plaque stability (Liu et al., 2015). Another two well-known pathways identified in our study were focal adhesion (hsa04510) and PI3K-Akt signaling pathway (hsa04151). Focal adhesion has an influence on both leucocyte motility within intima and interactions between platelets and endothelium through controlling cytoskeletal or adhesion dynamics (Charchar et al., 2012). Chan et al. (2014) employed integrative pathway and network analysis and revealed that focal adhesion was one of the shared molecular pathways for CAD and diabetes across diverse ethnicities. The PI3K-Akt pathway has been reported involved in cell proliferation, survival, and apoptosis. A two-stage systems genetics analysis of CAD GWAS data also found convincing association between PI3K-Akt pathway and CAD (Ghosh et al., 2015). Crosstalk analysis further demonstrated that these pathways might interconnect through overlapping genes instead of acting alone. Therefore, disruption or dysfunction of any key genes in the network may have cascading effects and result in a series of functional pathological consequences.

Our study is different from traditional fine-mapping approaches, which focus on identifying the causal variants that affect a trait of interest. Although very important, knowing which variants are causal does not equal to identifying the downstream effects of the variant on the trait. Moreover, if the causal genes affected by a locus are known, this can reduce the credible set of potential causal variants (Schaid et al., 2018; Broekema et al., 2020). Therefore, recent efforts in systems biology, integrating GWAS data and *a priori* knowledge from other omics, have focused on identifying such causal genes.

Unlike previous study (Braenne et al., 2015; Miller et al., 2016), which only focused on genome-wide significant region to predict causal genes, our study predicted plausible causal genes for CAD at genome-wide level by using genetic variants of both strong and subtle effects. Besides, to our knowledge, this is the first systematic analysis for CAD using comprehensive data from different layers to prioritize candidate genes. However, this study has several potential limitations. First, our study only paid attention to those genes that were prioritized by at least two computational methods. This strategy may omit some authentic CAD susceptibility genes. Besides, we assigned these methods with equal weights when combining the results. There might exist some discrepancies among the various methods because of different rationales and datasets. Further studies are needed to search the optimum weights. Second, we used eQTL data from peripheral blood samples when we conduced *Sherlock* and *SMR* analyses. Although blood tissue can be a proxy for eQTL effect in various diseases, it might be not ideal because we certainly lose power for eQTLs with tissue-specific effects (Supplementary Table S13). However, we gain power for genes with consistent effects across tissues because of the use of a very large sample size for eQTL analysis in blood. Future studies using large-scale eQTL data in pathophysiologically relevant tissues are needed to validate our results. Third, we did not consider other QTLs from different omic layers, such as DNA methylation (meQTLs), protein (pQTLs), or metabolites (mQTLs), which limited our ability to study from multi-omics perspective. In addition, the statistical methods did not perform causal inference; therefore, the genes identified in our study were only plausible candidate causal genes. However, the results still played a pivotal role in prioritizing genes for experimental follow-up. Further studies are needed to validate our results and elucidate the biological mechanisms.

## Conclusion

In summary, we integrated multi-dimensional data and depicted the landscape of plausible candidate causal genes for CAD. GO analysis further showed that these genes were enriched in lipid metabolism and extracellular region. Tissue-specific enrichment analysis revealed that these genes were significantly overexpressed in adipose and liver tissues. Further studies and experimental validations of these genes may shed light on mechanistic insights into CAD pathogenesis and provide potential drug targets for future therapeutics.

## Data Availability Statement

Our datasets analyzed during the current study were derived from the following public domain resources: Summary statistics of the GWAS is available from CARDIoGRAMplusC4D Consortium (http://www.cardiogramplusc4d.org/data-downloads/). Gene expression dataset is available from GTEx (https://gtexportal.org/home/). eQTL datasets are available from Jian Yang’s lab (https://cnsgenomics.com/software/smr/#Overview).

## Ethics Statement

The studies involving human participants were reviewed and approved by Peking University Institutional Review Board has evaluated the current protocol and found that no additional institutional review board approval was needed because the data are public available de-identified summary-level data. The patients/participants provided their written informed consent to participate in this study.

## Author Contributions

DC conceived the study, undertook project leadership, and are guarantors of this work. QZ wrote the first draft of the manuscript. QZ and YM analyzed the data and interpreted the data. QZ, YM, SC, and QC were involved in the data collection. All authors contributed to the drafting, critical revision of the manuscript, read and approved the final version of the manuscript.

## Conflict of Interest

The authors declare that the research was conducted in the absence of any commercial or financial relationships that could be construed as a potential conflict of interest.

## Abbreviations

CAD: coronary artery disease
CVD: cardiovascular disease
DAPPLE: Disease Association Protein–Protein Link Evaluator
DEPICT: Data-Driven Expression Prioritized Integration for Complex Traits
eQTL: expression quantitative trait locus
FDR: false discovery rate
GO: Gene Ontology
GTEx: Genotype-Tissue Expression
GWAB: Genome-Wide Association Boosting
GWAS: genome-wide association study
KEGG: Kyoto Encyclopedia of Genes and Genomes
LD: linkage disequilibrium
NetWAS: Network-Wide Association Study
PPI: protein–protein interaction
SMR: summary data-based Mendelian randomization
SNP: single-nucleotide polymorphism
TWAS: transcriptome-wide association studies.
