# Supplementary material for: The Integrated Landscape of Biological Candidate Causal Genes in Coronary Artery Disease
Source: Front Genet. 2020 Apr 21;11:320. doi: 10.3389/fgene.2020.00320 (PMC7186505; doi:10.3389/fgene.2020.00320)
Supplement: Supplementary file 1 [file Data_Sheet_1.docx]

**Supplementary Tables**

**Supplementary Table 1.** Causal genes identified by *Sherlock.*

| **Gene symbol** | **LBF ^a^** | **P-value ^b^** | **Supporting SNP ^c^** | **Location** | ***P* _eQTL_ ^d^** | ***P* _GWAS_ ^e^** |
| --- | --- | --- | --- | --- | --- | --- |
| PSRC1 | 7.58 | 1.74×10^-6^ | rs646776 (cis) | chr1:109620053 | 9.38×10^-9^ | 1.09×10^-23^ |
| NT5C2 | 7.46 | 1.74×10^-6^ | rs12217501 (cis) | chr10:104841879 | 3.73×10^-7^ | 1.54×10^-7^ |
| MRPS6 | 7.34 | 1.74×10^-6^ | rs7276739 (cis) | chr21:34510734 | 6.50×10^-6^ | 2.32×10^-8^ |
| MAT2A | 7.17 | 1.74×10^-6^ | rs7579665 (cis) | chr2:85596127 | 1.18×10^-6^ | 1.95×10^-9^ |
| LIPA | 7.07 | 1.74×10^-6^ | rs2246942 (cis) | chr10:90994866 | 4.84×10^-34^ | 3.51×10^-16^ |
| FES | 6.94 | 1.74×10^-6^ | rs1894400 (cis) | chr15:89229959 | 1.03×10^-8^ | 2.87×10^-12^ |
| GGCX | 6.78 | 1.74×10^-6^ | rs7568458 (cis) | chr2:85641686 | 3.09×10^-32^ | 2.39×10^-13^ |
| BSND | 6.75 | 1.74×10^-6^ | rs11206510 (cis) | chr1:55268627 | 4.53×10^-6^ | 3.68×10^-8^ |
| FAM177B | 6.45 | 1.74×10^-6^ | rs2068814 (cis) | chr1:220989624 | 6.88×10^-11^ | 2.66×10^-10^ |
| USP39 | 5.66 | 3.48×10^-6^ | rs2166529 (cis) | chr2:85595686 | 2.53×10^-6^ | 4.49×10^-12^ |
| CABIN1 | 5.32 | 3.48×10^-6^ | rs5760229 (cis) | chr22:22890254 | 7.26×10^-8^ | 3.25×10^-5^ |
| ABO | 5.30 | 3.48×10^-6^ | rs635634 (cis) | chr9:135144821 | 5.81×10^-15^ | 2.34×10^-12^ |
| SUSD2 | 5.22 | 6.96×10^-6^ | rs8141797 (cis) | chr22:22912041 | 7.89×10^-7^ | 3.11×10^-5^ |
| LPL | 5.17 | 6.96×10^-6^ | rs10096633 (cis) | chr8:19875201 | 8.95×10^-6^ | 8.95×10^-7^ |
| EIF6 | 5.02 | 6.96×10^-6^ | rs10485505 (cis) | chr20:32489228 | 1.47×10^-6^ | 1.72×10^-3^ |
| GALNT4 | 4.79 | 6.96×10^-6^ | rs11105325 (cis) | chr12:88462721 | 7.75×10^-6^ | 1.83×10^-7^ |

^a^ LBF (Log Bayes Factors for each gene) evaluates whether the combined evidence from GWAS and expression studies support a gene being associated with coronary artery disease. High Bayes factors suggests high probability that the gene is associated with coronary artery disease. For a given gene, individual LBF was calculated for each SNP and the sum of these constitutes the final LBF score for the gene. ^b^ P-value from Sherlock integrative analysis. ^c^ SNP with the highest LBF score. ^d^ P-value for eQTL SNP from the gene expression study (GTEx v7 whole blood). ^e^ P-value for eQTL SNP from the GWAS of coronary artery disease. It should be noted that some genes with different LBF scores may have the same P-values. This is because that Sherlock computes P-values by finite permutations. If the number of permutation is not extremely high, it is possible that two different values of LBF correspond to the same P-values (their rankings are the same).

**Supplementary Table 2.** Causal genes identified by *SMR.*

| **Gene**  **symbol** | **Probe ID** | **Top SNP** | **Location** | ***P* _eQTL_ ^a^** | ***P* _GWAS_ ^b^** | ***P* _SMR_ ^c^** | **P _HET_ ^d^** |
| --- | --- | --- | --- | --- | --- | --- | --- |
| PSRC1 | ILMN_1671843 | rs7528419 | chr1:109817192 | 3.40×10^-64^ | 3.77×10^-27^ | 9.02×10^-20^ | 0.212 |
| LIPA | ILMN_1718063 | rs1412444 | chr10:91002927 | 2.28×10^-276^ | 8.20×10^-14^ | 2.67×10^-13^ | 0.306 |
| SWAP70 | ILMN_1785175 | rs173396 | chr11:9759918 | 1.84×10^-69^ | 2.39×10^-8^ | 1.02×10^-7^ | 0.107 |
| NT5C2 | ILMN_1682165 | rs11191607 | chr10:104959188 | 1.04×10^-49^ | 3.00×10^-7^ | 1.26×10^-6^ | 0.106 |
| VAMP8 | ILMN_2190084 | rs2366639 | chr2:85809955 | 9.23×10^-196^ | 9.61×10^-12^ | 2.94×10^-11^ | 0.001 |
| MAT2A | ILMN_1737298 | rs7579665 | chr2:85742616 | 8.13×10^-14^ | 1.95×10^-9^ | 2.85×10^-6^ | 0.021 |

^a^ P-value for eQTL SNP from the gene expression study (CAGE et al).

^b^ P-value for eQTL SNP from the GWAS of coronary artery disease.

^c^ P-value from SMR integrative analysis.

^d^ P value of HEIDI test, only genes with P_HET_ > 0.05 were retained as plausible causal genes.

**Supplementary Table 3.** Causal genes identified by *DAPPLE*.

| **GENE** | **Region** | **P_corrected** |  | **GENE** | **Region** | **P_corrected** |
| --- | --- | --- | --- | --- | --- | --- |
| SVEP1 | 9q31.3 | 0.001997 |  | MRAS | 3q22.3 | 0.017901 |
| ZNF259 | 11q23.3 | 0.013937 |  | UMPS | 3q21.2 | 0.039561 |
| APOB | 2p24.1 | 0.001997 |  | C2 | 6p21.33 | 0.003992 |
| MIA3 | 1q41 | 0.009965 |  | FN1 | 2q35 | 0.001997 |
| HDAC9 | 7p21.1 | 0.01988 |  | LRP1 | 12q13.3 | 0.001997 |
| FES | 15q26.1 | 0.001997 |  | ZC3HC1 | 7q32.2 | 0.001997 |
| SLC22A3 | 6q25.3 | 0.005985 |  | FURIN | 15q26.1 | 0.003992 |
| NOS3 | 7q36.1 | 0.013937 |  | LPL | 8p21.3 | 0.001997 |
| SLC22A4 | 5q31.1 | 0.001997 |  | TGFB1 | 19q13.2 | 0.001997 |
| PLG | 6q26 | 0.001997 |  | CTR9 | 11p15.4 | 0.01592 |
| ABCG5 | 2p21 | 0.001997 |  | RHOA | 3p21.31 | 0.035641 |
| FLT1 | 13q12.3 | 0.001997 |  | IL6R | 1q21.3 | 0.037602 |
| HNF1A | 12q24.31 | 0.001997 |  | ANKS1A | 6p21.31 | 0.025805 |
| SH2B3 | 12q24.12 | 0.005985 |  | MC4R | 18q21.32 | 0.005985 |
| GGCX | 2p11.2 | 0.009965 |  | PMAIP1 | 18q21.32 | 0.01592 |
| LDLR | 19p13.2 | 0.001997 |  | SMG6 | 17p13.3 | 0.003992 |
| PDGFD | 11q22.3 | 0.003992 |  | SMAD3 | 15q22.33 | 0.017901 |
| UBE2Z | 17q21.32 | 0.001997 |  | VAMP8 | 2p11.2 | 0.049326 |
| APOA1 | 11q23.3 | 0.005985 |  | COL4A2 | 13q34 | 0.001997 |
| CETP | 16q13 | 0.001997 |  | ZEB2 | 2q22.3 | 0.003992 |
| PCSK9 | 1p32.3 | 0.001997 |  | SLC22A5 | 5q31.1 | 0.001997 |
| EDNRA | 4q31.22 | 0.007976 |  | TRIB1 | 8q24.13 | 0.005985 |
| ABCG8 | 2p21 | 0.001997 |  | PEMT | 17p11.2 | 0.01592 |
| REST | 4q12 | 0.035641 |  | SCARB1 | 12q24.31 | 0.001997 |
| COL4A1 | 13q34 | 0.001997 |  | CXCL12 | 10q11.21 | 0.001997 |
| SORT1 | 1p13.3 | 0.021857 |  | ARNTL | 11p15.3 | 0.001997 |
| GIGYF2 | 2q37.1 | 0.035641 |  | ITGB5 | 3q21.2 | 0.001997 |
| KSR2 | 12q24.23 | 0.007976 |  | LPA | 6q26 | 0.001997 |
| PPAP2B | 1p32.2 | 0.001997 |  | RASD1 | 17p11.2 | 0.005985 |

**Supplementary Table 4.** Gene Ontology (GO) analysis of causal genes prioritized by *DAPPLE*.

| **Category** | **Term** | **Count** | **%** | **P-Value** | **FDR** |
| --- | --- | --- | --- | --- | --- |
| BP | lipoprotein metabolic process | 8 | 14.3 | 3.20E-11 | 1.30E-08 |
| BP | cholesterol homeostasis | 9 | 16.1 | 3.00E-11 | 2.50E-08 |
| BP | cholesterol efflux | 5 | 8.9 | 1.20E-06 | 3.50E-04 |
| BP | cholesterol metabolic process | 6 | 10.7 | 2.80E-06 | 5.80E-04 |
| BP | transforming growth factor beta receptor signaling pathway | 6 | 10.7 | 1.20E-05 | 2.10E-03 |
| BP | cholesterol transport | 4 | 7.1 | 1.80E-05 | 2.50E-03 |
| BP | reverse cholesterol transport | 4 | 7.1 | 2.60E-05 | 3.10E-03 |
| BP | phospholipid transport | 4 | 7.1 | 7.30E-05 | 7.60E-03 |
| BP | triglyceride homeostasis | 4 | 7.1 | 8.20E-05 | 7.60E-03 |
| BP | quaternary ammonium group transport | 3 | 5.4 | 1.00E-04 | 8.70E-03 |
| BP | cholesterol import | 3 | 5.4 | 1.60E-04 | 1.20E-02 |
| BP | triglyceride metabolic process | 4 | 7.1 | 2.00E-04 | 1.40E-02 |
| BP | positive regulation of cholesterol storage | 3 | 5.4 | 2.20E-04 | 1.40E-02 |
| BP | low-density lipoprotein particle clearance | 3 | 5.4 | 2.90E-04 | 1.70E-02 |
| BP | receptor-mediated endocytosis | 6 | 10.7 | 3.50E-04 | 1.80E-02 |
| BP | intestinal cholesterol absorption | 3 | 5.4 | 3.70E-04 | 1.80E-02 |
| BP | negative regulation of fat cell differentiation | 4 | 7.1 | 3.50E-04 | 1.90E-02 |
| BP | phospholipid metabolic process | 4 | 7.1 | 7.30E-04 | 3.40E-02 |
| BP | lipoprotein transport | 3 | 5.4 | 1.10E-03 | 4.40E-02 |
| BP | high-density lipoprotein particle remodeling | 3 | 5.4 | 1.10E-03 | 4.40E-02 |
| BP | retinoid metabolic process | 4 | 7.1 | 1.00E-03 | 4.50E-02 |
| CC | cell surface | 11 | 19.6 | 4.40E-06 | 3.00E-04 |
| CC | receptor complex | 7 | 12.5 | 2.20E-06 | 3.00E-04 |
| CC | plasma membrane | 27 | 48.2 | 4.80E-05 | 2.20E-03 |
| CC | extracellular space | 14 | 25 | 1.40E-04 | 4.80E-03 |
| CC | extracellular region | 15 | 26.8 | 2.30E-04 | 6.10E-03 |
| CC | early endosome | 6 | 10.7 | 6.30E-04 | 1.40E-02 |
| CC | chylomicron | 3 | 5.4 | 8.00E-04 | 1.50E-02 |
| CC | apical plasma membrane | 6 | 10.7 | 1.80E-03 | 2.70E-02 |
| CC | very-low-density lipoprotein particle | 3 | 5.4 | 1.60E-03 | 2.70E-02 |
| CC | endoplasmic reticulum lumen | 5 | 8.9 | 2.70E-03 | 3.60E-02 |
| MF | apolipoprotein binding | 6 | 10.7 | 6.00E-10 | 1.30E-07 |
| MF | cholesterol transporter activity | 5 | 8.9 | 1.30E-07 | 1.50E-05 |
| MF | protein binding | 46 | 82.1 | 4.10E-06 | 3.00E-04 |
| MF | quaternary ammonium group transmembrane transporter activity | 3 | 5.4 | 1.00E-04 | 5.70E-03 |
| MF | low-density lipoprotein particle binding | 3 | 5.4 | 1.10E-03 | 4.60E-02 |
| MF | protease binding | 4 | 7.1 | 4.40E-03 | 1.50E-01 |
| MF | triglyceride binding | 2 | 3.6 | 6.50E-03 | 1.90E-01 |
| MF | cation antiporter activity | 2 | 3.6 | 6.50E-03 | 1.90E-01 |
| MF | carnitine transmembrane transporter activity | 2 | 3.6 | 9.70E-03 | 2.10E-01 |
| MF | apolipoprotein receptor binding | 2 | 3.6 | 9.70E-03 | 2.10E-01 |
| MF | serine-type endopeptidase activity | 5 | 8.9 | 9.50E-03 | 2.30E-01 |
| MF | lipoprotein transporter activity | 2 | 3.6 | 1.30E-02 | 2.50E-01 |
| MF | nerve growth factor binding | 2 | 3.6 | 1.60E-02 | 2.60E-01 |
| MF | heparin binding | 4 | 7.1 | 1.50E-02 | 2.70E-01 |
| MF | virus receptor activity | 3 | 5.4 | 2.20E-02 | 3.10E-01 |
| MF | high-density lipoprotein particle binding | 2 | 3.6 | 2.60E-02 | 3.40E-01 |
| MF | phospholipid transporter activity | 2 | 3.6 | 3.20E-02 | 3.80E-01 |
| MF | low-density lipoprotein receptor activity | 2 | 3.6 | 4.20E-02 | 4.40E-01 |
| MF | low-density lipoprotein particle receptor binding | 2 | 3.6 | 5.10E-02 | 4.90E-01 |

**Supplementary Table 5.** Causal genes identified by *NetWAS*.

| **Gene** | **Chromosome** | **Start** | **Stop** | **NetWAS score** |
| --- | --- | --- | --- | --- |
| SMC3 | 10 | 112327449 | 112364392 | 1.18809 |
| SNRPD3 | 22 | 24951618 | 24970932 | 1.12718 |
| PSMA7 | 20 | 60711783 | 60718514 | 1.09461 |
| DDX21 | 10 | 70715879 | 70744825 | 1.0373 |
| CDC27 | 17 | 45195310 | 45266665 | 0.989986 |
| NRP1 | 10 | 33466419 | 33623833 | 0.967821 |
| RPS2 | 16 | 2012059 | 2014827 | 0.965425 |
| CCT3 | 1 | 156278752 | 156308206 | 0.963875 |
| MAGOH | 1 | 53692564 | 53704282 | 0.92987 |
| CCT7 | 2 | 73461364 | 73480150 | 0.90232 |
| NFKB1 | 4 | 103422486 | 103538459 | 0.893698 |
| TMED2 | 12 | 124069076 | 124082688 | 0.887902 |
| AGPAT1 | 6 | 32135983 | 32145888 | 0.878021 |
| GORASP2 | 2 | 171784948 | 171823645 | 0.866176 |
| WDR77 | 1 | 111982512 | 111991915 | 0.861461 |
| SNRPA1 | 15 | 101821715 | 101835481 | 0.858185 |
| GSTM1 | 1 | 110230418 | 110240828 | 0.85748 |
| PSMA4 | 15 | 78832747 | 78841562 | 0.844837 |
| COA4 | 11 | 73583712 | 73587890 | 0.844771 |
| CCNH | 5 | 86687310 | 86708850 | 0.833245 |
| RRBP1 | 20 | 17594323 | 17662934 | 0.829347 |
| CDK4 | 12 | 58141510 | 58146304 | 0.822399 |
| PLK1 | 16 | 23690201 | 23701688 | 0.816082 |
| RPA1 | 17 | 1733273 | 1802848 | 0.808746 |
| LSM4 | 19 | 18417040 | 18434001 | 0.794894 |
| RNF13 | 3 | 149530475 | 149679926 | 0.794887 |
| EPRS | 1 | 220141940 | 220220000 | 0.781409 |
| HNRNPUL1 | 19 | 41768391 | 41813811 | 0.779672 |
| LSM7 | 19 | 2321520 | 2328614 | 0.771597 |
| COL4A2 | 13 | 110959631 | 111165374 | 0.769999 |
| PSMD11 | 17 | 30771481 | 30810337 | 0.75322 |
| CAP1 | 1 | 40505912 | 40538321 | 0.752337 |
| DNAJC8 | 1 | 28526789 | 28559542 | 0.748731 |
| FLOT2 | 17 | 27206356 | 27224715 | 0.747808 |
| EIF4G1 | 3 | 184032283 | 184053146 | 0.74549 |
| TRAM1 | 8 | 71485453 | 71520694 | 0.741021 |
| SNRPG | 2 | 70508510 | 70520869 | 0.73647 |
| PHACTR2 | 6 | 143929317 | 144152322 | 0.731234 |
| ELOVL1 | 1 | 43829068 | 43833745 | 0.724134 |
| PSMD4 | 1 | 151227176 | 151239955 | 0.722603 |
| ARHGDIA | 17 | 79825595 | 79829282 | 0.72227 |
| NOTCH2 | 1 | 120454176 | 120639880 | 0.719492 |
| DUSP5 | 10 | 112257625 | 112271302 | 0.718148 |
| RPL9 | 4 | 39455744 | 39460568 | 0.717524 |
| THBS1 | 15 | 39873280 | 39889668 | 0.716881 |
| EIF3E | 8 | 109213972 | 109260959 | 0.714904 |
| STIL | 1 | 47715811 | 47779819 | 0.713844 |
| UBA7 | 3 | 49842638 | 49851391 | 0.711209 |
| G3BP1 | 5 | 151151476 | 151184915 | 0.70017 |
| NDEL1 | 17 | 8316447 | 8371495 | 0.694589 |

**Supplementary Table 6.** Gene Ontology (GO) analysis of causal genes prioritized by *NetWAS*.

| **Category** | **Term** | **Count** | **%** | **P-Value** | **FDR** |
| --- | --- | --- | --- | --- | --- |
| BP | mRNA splicing, via spliceosome | 8 | 16 | 3.40E-06 | 5.60E-04 |
| BP | anaphase-promoting complex-dependent catabolic process | 6 | 12 | 3.30E-06 | 8.00E-04 |
| BP | positive regulation of ubiquitin-protein ligase activity involved in regulation of mitotic cell cycle transition | 6 | 12 | 2.70E-06 | 1.30E-03 |
| BP | NIK/NF-kappaB signaling | 5 | 10 | 4.00E-05 | 4.90E-03 |
| BP | negative regulation of ubiquitin-protein ligase activity involved in mitotic cell cycle | 5 | 10 | 5.40E-05 | 5.20E-03 |
| BP | spliceosomal snRNP assembly | 4 | 8 | 7.30E-05 | 5.90E-03 |
| BP | stimulatory C-type lectin receptor signaling pathway | 5 | 10 | 2.50E-04 | 1.50E-02 |
| BP | regulation of mRNA stability | 5 | 10 | 2.30E-04 | 1.60E-02 |
| BP | regulation of cellular amino acid metabolic process | 4 | 8 | 4.40E-04 | 1.90E-02 |
| BP | positive regulation of canonical Wnt signaling pathway | 5 | 10 | 4.10E-04 | 2.00E-02 |
| BP | nuclear-transcribed mRNA catabolic process, nonsense-mediated decay | 5 | 10 | 4.00E-04 | 2.10E-02 |
| BP | antigen processing and presentation of exogenous peptide antigen via MHC class I, TAP-dependent | 4 | 8 | 8.20E-04 | 3.30E-02 |
| BP | T cell receptor signaling pathway | 5 | 10 | 9.00E-04 | 3.30E-02 |
| BP | negative regulation of canonical Wnt signaling pathway | 5 | 10 | 1.30E-03 | 4.40E-02 |
| CC | cytosol | 29 | 58 | 1.20E-09 | 2.00E-07 |
| CC | nucleoplasm | 26 | 52 | 5.30E-09 | 4.30E-07 |
| CC | spliceosomal tri-snRNP complex | 4 | 8 | 1.80E-07 | 9.90E-06 |
| CC | catalytic step 2 spliceosome | 5 | 10 | 1.10E-04 | 4.40E-03 |
| CC | methylosome | 3 | 6 | 4.60E-04 | 1.50E-02 |
| CC | proteasome complex | 4 | 8 | 5.60E-04 | 1.50E-02 |
| CC | small nuclear ribonucleoprotein complex | 3 | 6 | 9.40E-04 | 2.20E-02 |
| CC | U2 snRNP | 3 | 6 | 1.30E-03 | 2.60E-02 |
| CC | chromatin | 4 | 8 | 1.80E-03 | 3.20E-02 |
| CC | U12-type spliceosomal complex | 3 | 6 | 2.20E-03 | 3.20E-02 |
| CC | spliceosomal complex | 4 | 8 | 2.10E-03 | 3.30E-02 |
| MF | protein binding | 45 | 90 | 6.10E-10 | 7.60E-08 |
| MF | poly(A) RNA binding | 14 | 28 | 8.30E-06 | 5.20E-04 |
| MF | U1 snRNP binding | 2 | 4 | 1.90E-02 | 3.90E-01 |
| MF | RNA binding | 6 | 12 | 1.80E-02 | 4.30E-01 |
| MF | U6 snRNA binding | 2 | 4 | 2.80E-02 | 4.40E-01 |

**Supplementary Table 7.** Causal genes identified by *Prix Fixe*.

| **Symbol** | **Chromosome** | **Region** | **PF Score** |
| --- | --- | --- | --- |
| PCSK9 | chr1 | p32.3 | 15 |
| DHCR24 | chr1 | p32.3 | 10 |
| SOD2 | chr6 | q25.3-q26 | 15 |
| LPA | chr6 | q25.3-q26 | 10 |
| PLG | chr6 | q25.3-q26 | 10 |
| MAP3K4 | chr6 | q25.3-q26 | 5 |
| CDKN2B | chr9 | p21.3 | 10 |
| CDKN2A | chr9 | p21.3 | 5 |
| ADORA2A | chr22 | q11.23 | 10 |
| DERL3 | chr22 | q11.23 | 5 |

PF score represented the original score without normalization.

**Supplementary Table 8.** Causal genes identified by Pavlides et al (*SMR*).

| **Gene**  **symbol** | **Probe ID** | **Top SNP** | **Location** | ***P* _eQTL_ ^a^** | ***P* _GWAS_ ^b^** | ***P* _SMR_ ^c^** | **P _HET_ ^d^** |
| --- | --- | --- | --- | --- | --- | --- | --- |
| VAMP8 | ILMN_2190084 | rs1058588 | chr2:85662382 | 1.78×10^-300^ | 1.02×10^-9^ | 1.42×10^-9^ | 0.17 |
| SWAP70 | ILMN_1785175 | rs360136 | chr11:9730093 | 9.34×10^-72^ | 8.05×10^-8^ | 2.74×10^-7^ | 0.43 |
| IL6R | ILMN_1696394 | rs4537545 | chr1:152685503 | 2.01×10^-29^ | 2.82×10^-7^ | 2.98×10^-6^ | 0.47 |
| ATP5G1 | ILMN_1712430 | rs1962412 | chr17:44325258 | 1.32×10^-44^ | 7.35×10^-7^ | 3.03×10^-6^ | 0.27 |
| EIF2B2 | ILMN_1713380 | rs175016 | chr14:74529386 | 1.78×10^-278^ | 4.72×10^-6^ | 5.63×10^-6^ | 0.23 |

^a^ P-value for eQTL SNP from the gene expression study (CAGE et al).

^b^ P-value for eQTL SNP from the GWAS of coronary artery disease.

^c^ P-value from SMR integrative analysis.

^d^ P value of HEIDI test, only genes with P_HET_ > 0.05 were retained as plausible causal genes.

**Supplementary Table 9.** Causal genes identified by Jung Eun Shim et al (*GWAB*).

| **Gene** | **Chromosome** | **Start** | **Stop** | **GWAB score** |
| --- | --- | --- | --- | --- |
| APP | 21 | 27252861 | 27543446 | 17.6715 |
| FN1 | 2 | 216225177 | 216300890 | 10.81164 |
| COL4A1 | 13 | 110801310 | 110959496 | 10.73739 |
| PTPN11 | 12 | 112856536 | 112947717 | 10.43329 |
| COL4A2 | 13 | 110959631 | 111165374 | 10.01686 |
| ACAD11 | 3 | 132276982 | 132378975 | 9.908116 |
| LPL | 8 | 19796582 | 19824770 | 9.609525 |
| CDK8 | 13 | 26828262 | 26979375 | 9.440382 |
| SPARC | 5 | 151040657 | 151066615 | 9.00873 |
| PECAM1 | 17 | 62396775 | 62404856 | 8.894338 |
| GUCY1B3 | 4 | 156680126 | 156728783 | 8.598617 |
| NPM1 | 5 | 170814652 | 170837888 | 8.548953 |
| MDM2 | 12 | 69201952 | 69239324 | 8.480917 |
| PLG | 6 | 161123225 | 161175086 | 8.411658 |
| COL5A2 | 2 | 189896641 | 190044668 | 8.244282 |
| FLT1 | 13 | 28874483 | 29069265 | 8.170113 |
| ESR1 | 6 | 152011631 | 152424409 | 8.055217 |
| ACAD10 | 12 | 112123857 | 112194911 | 8.015873 |
| PXN | 12 | 120648242 | 120703574 | 7.968733 |
| SMAD3 | 15 | 67358036 | 67487533 | 7.94423 |
| NME4 | 16 | 446752 | 450754 | 7.930827 |
| PPP1CC | 12 | 111157613 | 111180783 | 7.894355 |
| EGFR | 7 | 55086678 | 55279262 | 7.859982 |
| ATXN2 | 12 | 111890018 | 112037480 | 7.854726 |
| APOA5 | 11 | 116660086 | 116663136 | 7.641236 |
| IFNGR1 | 6 | 137518621 | 137540981 | 7.623709 |
| ASZ1 | 7 | 117003276 | 117067577 | 7.617983 |
| PTPN18 | 2 | 131113580 | 131132982 | 7.569061 |
| PARK2 | 6 | 161768590 | 163148834 | 7.505844 |
| CALD1 | 7 | 134464164 | 134655480 | 7.476911 |
| ALDH2 | 12 | 112204691 | 112247789 | 7.416813 |
| ABCG8 | 2 | 44066103 | 44105947 | 7.401296 |
| ABCG5 | 2 | 44039611 | 44066039 | 7.401296 |
| FBN1 | 15 | 48700503 | 48937985 | 7.343585 |
| CYP46A1 | 14 | 100150755 | 100193638 | 7.312923 |

**Supplementary Table 10.** Causal genes identified by van der Harst et al (*DEPICT*).

| **Gene symbol** | **Chromosome and position** | **GWAS P value** | **Nominal P value** | **False discovery rate** |
| --- | --- | --- | --- | --- |
| TGFB1 | chr19:41699115-41889988 | 2.36E-17 | 2.97E-27 | <=0.01 |
| SSH1 | chr12:109038885-109294819 | 3.31E-06 | 5.01E-22 | <=0.01 |
| FNDC3B | chr3:171757418-172119455 | 1.21E-09 | 1.25E-21 | <=0.01 |
| TRIM47 | chr17:73823310-73975515 | 5.33E-06 | 7.32E-20 | <=0.01 |
| ARAP3 | chr5:140710252-141061788 | 4.58E-06 | 9.76E-20 | <=0.01 |
| PTRF | chr17:40554468-40829048 | 2.33E-07 | 4.72E-19 | <=0.01 |
| TFPI | chr2:188207856-188430487 | 1.21E-07 | 1.59E-18 | <=0.01 |
| ADAMTSL4 | chr1:150521040-150968110 | 1.29E-09 | 2.25E-18 | <=0.01 |
| TGM2 | chr20:36756863-36794980 | 8.94E-06 | 2.95E-18 | <=0.01 |
| RAPH1 | chr2:202899310-204400133 | 1.29E-32 | 2.96E-18 | <=0.01 |
| VEGFA | chr6:43737921-43754224 | 4.00E-12 | 8.91E-18 | <=0.01 |
| GATA6 | chr18:19749404-19782491 | 1.49E-07 | 2.41E-17 | <=0.01 |
| C10orf26 | chr10:104503727-105206049 | 4.67E-15 | 2.69E-17 | <=0.01 |
| SMAD3 | chr15:67356101-67487533 | 5.68E-17 | 4.85E-17 | <=0.01 |
| AKAP13 | chr15:85777817-86338261 | 8.82E-07 | 5.77E-17 | <=0.01 |
| PVRL2 | chr19:45349393-45422606 | 2.14E-35 | 6.28E-17 | <=0.01 |
| FURIN | chr15:91411822-91538859 | 9.86E-27 | 7.08E-17 | <=0.01 |
| ZFP36L2 | chr2:43393800-43823185 | 1.28E-07 | 5.37E-16 | <=0.01 |
| NEDD9 | chr6:11183531-11382581 | 4.80E-07 | 1.67E-15 | <=0.01 |
| ETS2 | chr21:38437942-45746674 | 3.43E-07 | 2.27E-15 | <=0.01 |
| NFIB | chr9:14081842-14398982 | 8.77E-07 | 2.69E-15 | <=0.01 |
| DAB2 | chr5:39371780-39462402 | 5.30E-06 | 3.30E-15 | <=0.01 |
| IGF2R | chr6:160390131-161695093 | 9.78E-154 | 3.55E-15 | <=0.01 |
| AXL | chr19:41699115-41889988 | 2.36E-17 | 3.81E-15 | <=0.01 |
| HIC1 | chr17:1933431-2415200 | 4.11E-17 | 5.01E-15 | <=0.01 |
| KANK2 | chr19:11274944-11308243 | 1.57E-11 | 5.83E-15 | <=0.01 |
| SH2B3 | chr12:111374389-113336686 | 3.72E-27 | 1.13E-14 | <=0.01 |
| SERPINH1 | chr11:75273101-75283828 | 4.76E-11 | 1.21E-14 | <=0.01 |
| GALNT2 | chr1:230193536-230417870 | 6.40E-07 | 1.70E-14 | <=0.01 |
| ARNT | chr1:150521040-150968110 | 1.29E-09 | 3.32E-14 | <=0.01 |
| TNS1 | chr2:218664512-218867718 | 1.56E-12 | 3.84E-14 | <=0.01 |
| DUSP6 | chr12:89741050-89747048 | 7.43E-06 | 4.59E-14 | <=0.01 |
| MAP3K11 | chr11:65360326-65430565 | 2.29E-11 | 7.47E-14 | <=0.01 |
| NRP1 | chr10:33466420-33625190 | 2.47E-06 | 1.19E-13 | <=0.01 |
| COL4A2 | chr13:110801311-111165374 | 3.89E-23 | 1.24E-13 | <=0.01 |
| LDLR | chr19:11200038-11244492 | 1.93E-36 | 1.25E-13 | <=0.01 |
| FGD6 | chr12:95290831-95696566 | 1.06E-12 | 1.44E-13 | <=0.01 |
| LAMB2 | chr3:46742823-49894007 | 3.48E-11 | 1.58E-13 | <=0.01 |
| ADAMTS7 | chr15:78729773-79241916 | 5.48E-36 | 2.15E-13 | <=0.01 |
| COL4A1 | chr13:110801311-111165374 | 3.89E-23 | 3.06E-13 | <=0.01 |
| KLF3 | chr4:38665817-38702663 | 3.56E-07 | 3.31E-13 | <=0.01 |
| DAG1 | chr3:46742823-49894007 | 3.48E-11 | 3.53E-13 | <=0.01 |
| MAP3K1 | chr5:56111401-56267502 | 3.74E-08 | 3.95E-13 | <=0.01 |
| SFTPC | chr8:22014426-22069839 | 1.04E-11 | 4.51E-13 | <=0.01 |
| PDLIM5 | chr4:95373037-95589377 | 8.41E-07 | 4.58E-13 | <=0.01 |
| CALCRL | chr2:188207856-188430487 | 1.21E-07 | 7.92E-13 | <=0.01 |
| ST5 | chr11:8714898-8932498 | 7.78E-06 | 1.09E-12 | <=0.01 |
| TAGLN | chr11:116706467-117103241 | 4.06E-07 | 1.25E-12 | <=0.01 |
| BCAM | chr19:45281126-45324677 | 9.45E-07 | 1.25E-12 | <=0.01 |
| USP43 | chr17:9548950-9633001 | 4.30E-06 | 1.32E-12 | <=0.01 |
| NFAT5 | chr16:69598997-70472991 | 3.62E-07 | 1.43E-12 | <=0.01 |
| BMP1 | chr8:22014426-22069839 | 1.04E-11 | 1.73E-12 | <=0.01 |
| KLHDC8B | chr3:46742823-49894007 | 3.48E-11 | 1.76E-12 | <=0.01 |
| PDLIM7 | chr5:176910395-176981542 | 1.56E-06 | 1.84E-12 | <=0.01 |
| MTMR3 | chr22:30279144-31521442 | 2.00E-07 | 2.39E-12 | <=0.01 |
| REST | chr4:57774042-57931769 | 1.17E-08 | 3.40E-12 | <=0.01 |
| MAFB | chr20:39314488-39317880 | 5.73E-07 | 5.56E-12 | <=0.01 |
| ST3GAL2 | chr16:69598997-70472991 | 3.62E-07 | 5.85E-12 | <=0.01 |
| CXCL12 | chr10:44787706-44881941 | 3.50E-24 | 6.91E-12 | <=0.01 |
| SH3PXD2A | chr10:105348285-105677963 | 9.63E-10 | 1.01E-11 | <=0.01 |
| PTPN23 | chr3:46742823-49894007 | 3.48E-11 | 1.07E-11 | <=0.01 |
| MERTK | chr2:112656056-112945791 | 2.62E-07 | 1.08E-11 | <=0.01 |
| PFKFB4 | chr3:46742823-49894007 | 3.48E-11 | 1.18E-11 | <=0.01 |
| PRKAR2A | chr3:46742823-49894007 | 3.48E-11 | 1.19E-11 | <=0.01 |
| ALS2CL | chr3:46710487-46735194 | 1.10E-08 | 1.29E-11 | <=0.01 |
| TAB2 | chr6:149539777-149806197 | 1.28E-07 | 1.58E-11 | <=0.01 |
| EXOC3L4 | chr14:103566481-103576896 | 9.70E-06 | 1.64E-11 | <=0.01 |
| RAB27B | chr18:52495708-52562747 | 2.60E-06 | 1.68E-11 | <=0.01 |
| C5orf41 | chr5:172483355-172591390 | 6.99E-06 | 2.29E-11 | <=0.01 |
| C17orf57 | chr17:45400656-45789427 | 1.69E-06 | 2.41E-11 | <=0.01 |
| CDC42SE1 | chr1:150980896-151098018 | 3.28E-06 | 2.55E-11 | <=0.01 |
| GGT5 | chr22:24615622-24924358 | 3.73E-09 | 3.02E-11 | <=0.01 |
| COL6A3 | chr2:238232646-238323018 | 2.64E-08 | 3.35E-11 | <=0.01 |
| FN1 | chr2:216225163-216300895 | 1.58E-13 | 3.39E-11 | <=0.01 |
| SLC44A2 | chr19:10713121-11172958 | 5.36E-16 | 3.47E-11 | <=0.01 |
| ZEB2 | chr2:145121063-145282147 | 2.76E-09 | 3.94E-11 | <=0.01 |
| SMAD7 | chr18:46446223-46477081 | 8.95E-09 | 4.86E-11 | <=0.01 |
| OAZ2 | chr15:64679947-65067786 | 3.90E-08 | 5.08E-11 | <=0.01 |
| PALLD | chr4:169418217-169849608 | 3.09E-08 | 5.55E-11 | <=0.01 |
| ANGPTL4 | chr19:8429011-8439257 | 3.57E-10 | 5.66E-11 | <=0.01 |
| MKL1 | chr22:40806285-41032706 | 4.40E-08 | 6.11E-11 | <=0.01 |
| NGF | chr1:115828539-115880857 | 2.02E-08 | 7.03E-11 | <=0.01 |
| GEM | chr8:95261481-95274578 | 8.47E-07 | 7.43E-11 | <=0.01 |
| CDKN2B | chr9:21802635-22121096 | 8.82E-223 | 7.61E-11 | <=0.01 |
| RELA | chr11:65360326-65430565 | 2.29E-11 | 9.97E-11 | <=0.01 |
| NOS3 | chr7:150688083-150711676 | 1.35E-20 | 1.07E-10 | <=0.01 |
| RGL3 | chr19:11466062-11530018 | 4.09E-08 | 1.09E-10 | <=0.01 |
| PROCR | chr20:32868074-33880204 | 6.84E-12 | 1.29E-10 | <=0.01 |
| CTTN | chr11:70116806-70282690 | 8.32E-07 | 1.30E-10 | <=0.01 |
| DNM2 | chr19:10713121-11172958 | 5.36E-16 | 1.44E-10 | <=0.01 |
| SHISA5 | chr3:46742823-49894007 | 3.48E-11 | 1.69E-10 | <=0.01 |
| PPAP2B | chr1:56960419-57110974 | 8.04E-28 | 1.85E-10 | <=0.01 |
| NBEAL1 | chr2:202899310-204400133 | 1.29E-32 | 2.29E-10 | <=0.01 |
| LOX | chr5:121297656-121414206 | 6.03E-08 | 2.58E-10 | <=0.01 |
| KIAA1217 | chr10:23983675-24836772 | 2.54E-07 | 2.73E-10 | <=0.01 |
| ARHGAP42 | chr11:100558384-100861656 | 6.79E-11 | 4.05E-10 | <=0.01 |
| PPFIA1 | chr11:70116806-70282690 | 8.32E-07 | 4.22E-10 | <=0.01 |
| KLF4 | chr9:110247133-110252763 | 3.59E-12 | 4.22E-10 | <=0.01 |
| TBC1D10A | chr22:30279144-31521442 | 2.00E-07 | 4.86E-10 | <=0.01 |
| RAB5C | chr17:40253422-40307035 | 7.36E-09 | 5.33E-10 | <=0.01 |
| ZNF609 | chr15:64679947-65067786 | 3.90E-08 | 5.53E-10 | <=0.01 |
| RBPMS2 | chr15:64679947-65067786 | 3.90E-08 | 5.83E-10 | <=0.01 |
| C3orf54 | chr3:46742823-49894007 | 3.48E-11 | 6.04E-10 | <=0.01 |
| CBLC | chr19:45281126-45324677 | 9.45E-07 | 6.37E-10 | <=0.01 |
| LAYN | chr11:111338251-111751967 | 7.75E-07 | 7.21E-10 | <=0.01 |
| LNPEP | chr5:96149731-96373219 | 2.79E-06 | 7.31E-10 | <=0.01 |
| LOXL1 | chr15:74218792-74244478 | 1.23E-06 | 7.53E-10 | <=0.01 |
| TRIB1 | chr8:126442563-126450647 | 7.67E-23 | 1.59E-09 | <=0.01 |
| MAST4 | chr5:65892176-66465423 | 6.69E-07 | 1.63E-09 | <=0.01 |
| RRBP1 | chr20:17474550-17662940 | 2.66E-07 | 1.92E-09 | <=0.01 |
| NBEAL2 | chr3:46742823-49894007 | 3.48E-11 | 2.07E-09 | <=0.01 |
| NRIP1 | chr21:16333556-16437321 | 8.82E-08 | 2.33E-09 | <=0.01 |
| BACH1 | chr21:30449792-31009660 | 4.16E-09 | 2.39E-09 | <=0.01 |
| EDN1 | chr6:12290596-12297427 | 1.36E-07 | 2.40E-09 | <=0.01 |
| MYBPC3 | chr11:47352957-48192393 | 1.90E-06 | 2.63E-09 | <=0.01 |
| TEAD3 | chr6:35441374-35696360 | 4.84E-08 | 2.85E-09 | <=0.01 |
| REEP3 | chr10:65281123-65384883 | 2.19E-06 | 3.08E-09 | <=0.01 |
| PTGIS | chr20:48123992-48184683 | 2.04E-06 | 3.28E-09 | <=0.01 |
| APOB | chr2:21224301-21266945 | 5.74E-17 | 3.70E-09 | <=0.01 |
| CSNK1G2 | chr19:1905371-2015702 | 6.73E-06 | 4.06E-09 | <=0.01 |
| TBX3 | chr12:115108059-115121969 | 1.37E-06 | 4.84E-09 | <=0.01 |
| ABCA8 | chr17:66863433-66951533 | 9.51E-07 | 5.17E-09 | <=0.01 |
| PLTP | chr20:44527399-44600833 | 4.40E-09 | 5.96E-09 | <=0.01 |
| MYO9B | chr19:17160573-17324103 | 1.09E-07 | 6.66E-09 | <=0.01 |
| APOA1 | chr11:116706467-117103241 | 4.06E-07 | 8.38E-09 | <=0.01 |
| SBF2 | chr11:9685624-10315754 | 6.26E-12 | 8.49E-09 | <=0.01 |
| COL4A4 | chr2:227700297-228028829 | 8.24E-06 | 8.60E-09 | <=0.01 |
| CPD | chr17:27900487-28853837 | 2.90E-10 | 9.61E-09 | <=0.01 |
| PLXNB1 | chr3:46742823-49894007 | 3.48E-11 | 9.78E-09 | <=0.01 |
| ABCA1 | chr9:107543283-107690518 | 4.00E-07 | 1.04E-08 | <=0.01 |
| ANKRD50 | chr4:125585207-125633887 | 2.22E-06 | 1.05E-08 | <=0.01 |
| C17orf63 | chr17:26989302-27188085 | 4.55E-06 | 1.12E-08 | <=0.01 |
| TCF21 | chr6:134210276-134216691 | 3.56E-31 | 1.26E-08 | <=0.01 |
| ADAM19 | chr5:156693089-157002783 | 9.56E-06 | 1.30E-08 | <=0.01 |
| SKI | chr1:2160134-2241558 | 1.33E-08 | 1.65E-08 | <=0.01 |
| TSPAN14 | chr10:82031576-82282387 | 1.72E-11 | 1.65E-08 | <=0.01 |
| ITGB5 | chr3:123798870-124606674 | 1.45E-14 | 2.03E-08 | <=0.01 |
| CDKN1A | chr6:36555311-36932613 | 3.48E-08 | 2.10E-08 | <=0.01 |
| GJA1 | chr6:121756791-121770873 | 8.85E-06 | 2.51E-08 | <=0.01 |
| ST3GAL4 | chr11:126081309-126310239 | 2.00E-07 | 2.72E-08 | <=0.01 |
| DISP1 | chr1:222988406-223179337 | 8.55E-06 | 3.27E-08 | <=0.01 |
| RSPO3 | chr6:127439749-127518910 | 1.96E-06 | 3.76E-08 | <=0.01 |
| PKN2 | chr1:89149905-89357627 | 1.68E-06 | 3.83E-08 | <=0.01 |
| EDNRA | chr4:148402069-148466106 | 5.18E-24 | 3.87E-08 | <=0.01 |
| DIAPH1 | chr5:140710252-141061788 | 4.58E-06 | 3.88E-08 | <=0.01 |
| ZBTB46 | chr20:62375019-62462597 | 5.56E-07 | 4.20E-08 | <=0.01 |
| C1S | chr12:7096351-7178336 | 6.15E-10 | 4.64E-08 | <=0.01 |
| FAM109A | chr12:111374389-113336686 | 3.72E-27 | 5.35E-08 | <=0.01 |
| PLAUR | chr19:44150271-44174502 | 1.81E-06 | 5.49E-08 | <=0.01 |
| RAB34 | chr17:26989302-27188085 | 4.55E-06 | 5.54E-08 | <=0.01 |
| SSH2 | chr17:27900487-28853837 | 2.90E-10 | 5.55E-08 | <=0.01 |
| SCAMP2 | chr15:75041184-75230509 | 2.41E-06 | 5.66E-08 | <=0.01 |
| SCAP | chr3:46742823-49894007 | 3.48E-11 | 5.89E-08 | <=0.01 |
| CSK | chr15:75041184-75230509 | 2.41E-06 | 5.96E-08 | <=0.01 |
| BCAR1 | chr16:75237994-75529282 | 1.90E-16 | 6.11E-08 | <=0.01 |
| PDE5A | chr4:120056939-120550146 | 1.27E-08 | 7.89E-08 | <=0.01 |
| FLT1 | chr13:28874489-29069265 | 5.36E-11 | 8.69E-08 | <=0.01 |
| OTUD7B | chr1:149895209-149982686 | 1.84E-06 | 1.15E-07 | <=0.01 |
| MAGI3 | chr1:113933371-114414381 | 6.60E-07 | 1.20E-07 | <=0.01 |
| DDIT4 | chr10:73856278-74647452 | 9.40E-06 | 1.29E-07 | <=0.01 |
| TMEM204 | chr16:1543364-1662109 | 1.83E-07 | 1.29E-07 | <=0.01 |
| GDF15 | chr19:18451408-18508415 | 1.32E-07 | 1.30E-07 | <=0.01 |
| CABIN1 | chr22:24407642-24585078 | 1.95E-07 | 1.63E-07 | <=0.01 |
| PPARG | chr3:12328867-12475855 | 4.40E-06 | 1.66E-07 | <=0.01 |
| VAMP5 | chr2:85766288-85876406 | 1.81E-23 | 1.71E-07 | <=0.01 |
| CTSH | chr15:78729773-79241916 | 5.48E-36 | 1.74E-07 | <=0.01 |
| RYBP | chr3:72423744-72496069 | 4.24E-06 | 1.80E-07 | <=0.01 |
| RCN3 | chr19:50030875-50046889 | 9.64E-06 | 1.82E-07 | <=0.01 |
| ARL15 | chr5:53179775-53606412 | 2.01E-07 | 1.89E-07 | <=0.01 |
| SPECC1L | chr22:24615622-24924358 | 3.73E-09 | 2.11E-07 | <=0.01 |
| ELL | chr19:18553475-18688269 | 3.02E-13 | 2.81E-07 | <=0.01 |
| PID1 | chr2:229715242-230136001 | 1.47E-06 | 3.16E-07 | <=0.01 |
| TGFB2 | chr1:218519577-218617961 | 3.16E-06 | 3.25E-07 | <=0.01 |
| CDH13 | chr16:82660663-83829174 | 1.61E-16 | 3.85E-07 | <=0.01 |
| MFSD10 | chr4:2845584-3042474 | 5.55E-06 | 3.99E-07 | <=0.01 |
| MCL1 | chr1:150521040-150968110 | 1.29E-09 | 4.52E-07 | <=0.01 |
| PLEKHH2 | chr2:43864412-44105605 | 1.84E-18 | 5.11E-07 | <=0.01 |
| SLC39A13 | chr11:47352957-48192393 | 1.90E-06 | 6.20E-07 | <=0.01 |
| ABHD2 | chr15:89631405-89745591 | 5.21E-11 | 6.44E-07 | <=0.01 |
| KLHL25 | chr15:85777817-86338261 | 8.82E-07 | 6.71E-07 | <=0.01 |
| LGR4 | chr11:27387508-27899195 | 6.36E-06 | 6.80E-07 | <=0.01 |
| NCOA6 | chr20:32868074-33880204 | 6.84E-12 | 6.98E-07 | <=0.01 |
| KDM4B | chr19:4909510-5153606 | 5.63E-06 | 7.01E-07 | <=0.01 |
| CETP | chr16:56995762-57017757 | 1.19E-09 | 7.60E-07 | <=0.01 |
| AKAP12 | chr6:151561134-151679692 | 1.73E-06 | 7.85E-07 | <=0.01 |
| TCF7L2 | chr10:114710009-114927437 | 8.69E-07 | 8.20E-07 | <=0.01 |
| ZBTB20 | chr3:114056941-114866118 | 8.77E-06 | 8.53E-07 | <=0.01 |
| MITF | chr3:69788586-70017488 | 5.39E-07 | 8.77E-07 | <=0.01 |
| IGF2BP2 | chr3:185361527-185542844 | 4.93E-07 | 9.23E-07 | <=0.01 |
| WNT2 | chr7:116916685-116963343 | 1.04E-06 | 9.38E-07 | <=0.01 |
| NPHP3 | chr3:132136370-132441303 | 3.00E-09 | 9.44E-07 | <=0.01 |
| DYSF | chr2:71680852-71913898 | 1.22E-06 | 9.59E-07 | <=0.01 |
| SERPINA1 | chr14:94843084-94857030 | 8.44E-10 | 9.94E-07 | <=0.01 |
| FZD7 | chr2:202899310-204400133 | 1.29E-32 | 1.00E-06 | <=0.01 |
| BMPR2 | chr2:202899310-204400133 | 1.29E-32 | 1.09E-06 | <=0.01 |
| SHROOM3 | chr4:77135193-77704406 | 1.64E-10 | 1.13E-06 | <=0.01 |
| MPZL2 | chr11:118064455-118443685 | 2.01E-06 | 1.14E-06 | <=0.01 |
| TMEM150A | chr2:85766288-85876406 | 1.81E-23 | 1.17E-06 | <=0.01 |
| SASH1 | chr6:148593440-148873186 | 5.17E-06 | 1.19E-06 | <=0.01 |
| TRAF4 | chr17:26989302-27188085 | 4.55E-06 | 1.21E-06 | <=0.01 |
| IGF2BP1 | chr17:47074774-47133012 | 1.46E-11 | 1.30E-06 | <=0.01 |
| KIAA0040 | chr1:175036994-175162079 | 1.62E-06 | 1.35E-06 | <=0.01 |
| ZFHX3 | chr16:72042643-73093597 | 2.92E-11 | 1.36E-06 | <=0.01 |
| SMG6 | chr17:1933431-2415200 | 4.11E-17 | 1.63E-06 | <=0.01 |
| TSPAN9 | chr12:3186521-3395730 | 4.94E-06 | 1.80E-06 | <=0.01 |
| SLC26A6 | chr3:46742823-49894007 | 3.48E-11 | 2.05E-06 | <=0.01 |
| GXYLT2 | chr3:72798428-73047289 | 8.23E-06 | 2.11E-06 | <=0.01 |
| HTRA1 | chr10:124221041-124274424 | 8.02E-11 | 2.27E-06 | <=0.01 |
| MYL3 | chr3:46742823-49894007 | 3.48E-11 | 2.40E-06 | <=0.01 |
| MYO7B | chr2:128293378-128568761 | 8.78E-06 | 2.72E-06 | <=0.01 |
| MIER3 | chr5:56111401-56267502 | 3.74E-08 | 2.76E-06 | <=0.01 |
| PCSK9 | chr1:55505221-55530525 | 1.86E-22 | 2.94E-06 | <=0.01 |
| MLL | chr11:118064455-118443685 | 2.01E-06 | 3.02E-06 | <=0.01 |
| SCAMP4 | chr19:1905371-2015702 | 6.73E-06 | 3.08E-06 | <=0.01 |
| MTMR11 | chr1:149895209-149982686 | 1.84E-06 | 3.35E-06 | <=0.01 |
| PCNXL3 | chr11:65360326-65430565 | 2.29E-11 | 3.72E-06 | <=0.01 |
| NEK9 | chr14:75469614-75643334 | 3.60E-10 | 3.74E-06 | <=0.01 |
| COL7A1 | chr3:46742823-49894007 | 3.48E-11 | 3.89E-06 | <=0.01 |
| RAC1 | chr7:6369040-6523873 | 2.10E-08 | 3.93E-06 | <=0.01 |
| PTK7 | chr6:43044006-43129457 | 1.31E-07 | 3.94E-06 | <=0.01 |
| PCDHGA10 | chr5:140710252-141061788 | 4.58E-06 | 4.25E-06 | <=0.01 |
| ALCAM | chr3:105085753-105295744 | 6.80E-06 | 4.92E-06 | <=0.01 |
| LIMS2 | chr2:128293378-128568761 | 8.78E-06 | 5.19E-06 | <=0.01 |
| AHDC1 | chr1:27860546-27961788 | 1.85E-06 | 5.31E-06 | <=0.01 |
| ACAA2 | chr18:47309869-47340273 | 1.14E-09 | 5.95E-06 | <=0.01 |
| PLEKHA7 | chr11:16799842-17074583 | 1.07E-07 | 5.97E-06 | <=0.01 |
| P4HA2 | chr5:131527531-131811736 | 4.74E-10 | 6.26E-06 | <=0.01 |
| SREBF1 | chr17:17408877-18083116 | 9.51E-10 | 6.42E-06 | <=0.01 |
| LPL | chr8:19759228-19824770 | 5.15E-14 | 7.42E-06 | <=0.01 |
| ZNF787 | chr19:56598732-56632649 | 4.20E-06 | 7.45E-06 | <=0.01 |
| RASGEF1B | chr4:82347547-82393082 | 2.49E-10 | 7.51E-06 | <=0.01 |
| WWP2 | chr16:69598997-70472991 | 3.62E-07 | 7.58E-06 | <=0.01 |
| C5 | chr9:123664671-123837452 | 8.82E-08 | 7.72E-06 | <=0.01 |
| LPA | chr6:160390131-161695093 | 9.78E-154 | 8.25E-06 | <=0.01 |
| PLCG1 | chr20:39657458-39946312 | 1.12E-08 | 8.34E-06 | <=0.01 |
| CARM1 | chr19:10713121-11172958 | 5.36E-16 | 8.51E-06 | <=0.01 |
| DENND5A | chr11:9160372-9336327 | 6.36E-06 | 8.85E-06 | <=0.01 |
| CLPTM1 | chr19:45445495-45541452 | 1.53E-06 | 8.98E-06 | <=0.01 |
| LHFPL2 | chr5:77638189-78065844 | 9.03E-06 | 9.37E-06 | <=0.01 |
| RAI1 | chr17:17408877-18083116 | 9.51E-10 | 9.71E-06 | <=0.01 |
| FGD5 | chr3:14860469-15140670 | 3.21E-11 | 1.02E-05 | <=0.01 |
| GIGYF2 | chr2:233562009-233877982 | 1.15E-06 | 1.04E-05 | <=0.01 |
| CYP2S1 | chr19:41699115-41889988 | 2.36E-17 | 1.05E-05 | <=0.01 |
| APOC3 | chr11:116618886-116703788 | 7.03E-13 | 1.06E-05 | <=0.01 |
| SCUBE3 | chr6:35085848-35220856 | 1.62E-10 | 1.09E-05 | <=0.01 |
| ZC3H12A | chr1:37920480-38032458 | 2.34E-06 | 1.15E-05 | <=0.01 |
| PRDM6 | chr5:122422943-122523745 | 9.32E-07 | 1.15E-05 | <=0.01 |
| TRIM4 | chr7:99425636-99573780 | 1.70E-06 | 1.19E-05 | <=0.01 |
| ITCH | chr20:32868074-33880204 | 6.84E-12 | 1.24E-05 | <=0.01 |
| GATAD2A | chr19:19322782-19774502 | 3.18E-07 | 1.32E-05 | <=0.01 |
| C1GALT1 | chr7:7196565-7288251 | 4.94E-07 | 1.39E-05 | <=0.01 |
| SETD2 | chr3:46742823-49894007 | 3.48E-11 | 1.42E-05 | <=0.01 |
| BCL3 | chr19:45250962-45263301 | 1.91E-09 | 1.53E-05 | <=0.01 |
| MSL2 | chr3:135684515-136729927 | 6.08E-15 | 1.57E-05 | <=0.01 |
| ACVR2A | chr2:148602086-148688393 | 3.39E-07 | 1.62E-05 | <=0.01 |
| OGDH | chr7:44605016-44748665 | 1.72E-06 | 1.62E-05 | <=0.01 |
| ARL4C | chr2:235401685-235405697 | 5.56E-08 | 1.81E-05 | <=0.01 |
| LIPG | chr18:47088427-47119278 | 1.27E-06 | 1.85E-05 | <=0.01 |
| CERS2 | chr1:150521040-150968110 | 1.29E-09 | 1.91E-05 | <=0.01 |
| PDE3A | chr12:20522179-20837315 | 2.86E-14 | 1.92E-05 | <=0.01 |
| C21orf116 | chr21:16333556-16437321 | 8.82E-08 | 1.96E-05 | <=0.01 |
| DOCK5 | chr8:25042238-25275598 | 4.11E-07 | 2.14E-05 | <=0.01 |
| CSNK1G3 | chr5:122680579-122952739 | 2.57E-07 | 2.15E-05 | <=0.01 |
| VAT1 | chr17:41166622-41466266 | 8.40E-07 | 2.22E-05 | <=0.01 |
| EYA3 | chr1:28296855-28415207 | 7.20E-06 | 2.23E-05 | <=0.01 |
| HYOU1 | chr11:118914899-118964259 | 2.88E-07 | 2.27E-05 | <=0.01 |
| PTPRJ | chr11:47352957-48192393 | 1.90E-06 | 2.29E-05 | <=0.01 |
| DSC3 | chr18:28570052-28622781 | 9.35E-06 | 2.69E-05 | <=0.01 |
| SPI1 | chr11:47352957-48192393 | 1.90E-06 | 3.15E-05 | <=0.01 |
| FGR | chr1:27860546-27961788 | 1.85E-06 | 3.34E-05 | <=0.01 |
| HBP1 | chr7:106685094-107358254 | 4.53E-08 | 3.61E-05 | <=0.01 |
| SWAP70 | chr11:9685624-10315754 | 6.26E-12 | 3.64E-05 | <=0.01 |
| KDELR2 | chr7:6369040-6523873 | 2.10E-08 | 4.06E-05 | <=0.01 |
| CORO1C | chr12:109038885-109294819 | 3.31E-06 | 4.30E-05 | <=0.01 |
| ATF3 | chr1:212738676-212794119 | 8.18E-06 | 4.72E-05 | <=0.01 |
| SLC27A6 | chr5:127593601-128369335 | 1.41E-06 | 4.72E-05 | <=0.01 |
| PLEKHH3 | chr17:40554468-40829048 | 2.33E-07 | 4.82E-05 | <=0.01 |
| TXNDC5 | chr6:7881483-8026646 | 2.60E-06 | 5.13E-05 | <=0.01 |
| PCGF2 | chr17:36890150-36981589 | 5.37E-06 | 5.39E-05 | <=0.01 |
| TNN | chr1:175036994-175162079 | 1.62E-06 | 5.46E-05 | <=0.01 |
| SIK2 | chr11:111338251-111751967 | 7.75E-07 | 5.86E-05 | <=0.01 |
| ACOX1 | chr17:73823310-73975515 | 5.33E-06 | 5.91E-05 | <=0.01 |
| FHL3 | chr1:38326369-38471278 | 4.13E-10 | 6.44E-05 | <=0.01 |
| TEK | chr9:27109139-27230173 | 5.32E-07 | 6.89E-05 | <=0.01 |
| CCDC97 | chr19:41699115-41889988 | 2.36E-17 | 7.38E-05 | <=0.01 |
| EPOR | chr19:11466062-11530018 | 4.09E-08 | 7.41E-05 | <=0.01 |
| ATXN2 | chr12:111374389-113336686 | 3.72E-27 | 7.89E-05 | <=0.01 |
| PRUNE | chr1:150980896-151098018 | 3.28E-06 | 8.50E-05 | <=0.01 |
| PLCE1 | chr10:95753746-96088149 | 1.06E-06 | 8.52E-05 | <=0.01 |
| SLC22A1 | chr6:160390131-161695093 | 9.78E-154 | 8.71E-05 | <=0.01 |
| APOE | chr19:45349393-45422606 | 2.14E-35 | 8.97E-05 | <=0.01 |
| SPAG9 | chr17:49039535-49198226 | 5.56E-06 | 9.17E-05 | <=0.01 |
| APOC1 | chr19:45349393-45422606 | 2.14E-35 | 9.17E-05 | <=0.01 |
| FAM46A | chr6:82201156-82523874 | 6.58E-10 | 9.57E-05 | <=0.01 |
| DSTN | chr20:17474550-17662940 | 2.66E-07 | 9.68E-05 | <=0.01 |
| PCIF1 | chr20:44527399-44600833 | 4.40E-09 | 9.71E-05 | <=0.01 |
| ATN1 | chr12:6980099-7051484 | 5.91E-06 | 1.08E-04 | <=0.01 |
| ALDH2 | chr12:111374389-113336686 | 3.72E-27 | 1.09E-04 | <=0.01 |
| FBN2 | chr5:127593601-128369335 | 1.41E-06 | 1.14E-04 | <=0.01 |
| FBXL20 | chr17:37408897-37557898 | 9.79E-06 | 1.22E-04 | <=0.01 |
| ZNF335 | chr20:44527399-44600833 | 4.40E-09 | 1.29E-04 | <=0.01 |
| SIDT2 | chr11:116706467-117103241 | 4.06E-07 | 1.30E-04 | <=0.01 |
| APOF | chr12:56754353-56756607 | 4.56E-06 | 1.31E-04 | <=0.01 |
| USP4 | chr3:46742823-49894007 | 3.48E-11 | 1.31E-04 | <=0.01 |
| CX3CL1 | chr16:57392684-57449974 | 8.42E-06 | 1.34E-04 | <=0.01 |
| HP | chr16:72042643-73093597 | 2.92E-11 | 1.37E-04 | <=0.01 |
| DNAJB12 | chr10:73856278-74647452 | 9.40E-06 | 1.40E-04 | <=0.01 |
| ACVRL1 | chr12:52281744-52390862 | 2.73E-06 | 1.42E-04 | <=0.01 |
| ZNF652 | chr17:47280153-47439835 | 1.63E-15 | 1.56E-04 | <=0.01 |
| FAM60A | chr12:31433518-31479992 | 4.57E-06 | 1.58E-04 | <=0.01 |
| HNRNPUL1 | chr19:41699115-41889988 | 2.36E-17 | 1.64E-04 | <=0.01 |
| EFNA5 | chr5:106712590-107006596 | 1.05E-07 | 1.70E-04 | <=0.01 |
| MPDZ | chr9:13105703-13279589 | 2.21E-07 | 1.76E-04 | <=0.01 |
| LIPC | chr15:58702768-58861151 | 7.24E-06 | 1.82E-04 | <=0.01 |
| TBX20 | chr7:35242042-35293758 | 8.34E-08 | 1.85E-04 | <=0.01 |
| SOD2 | chr6:160100096-160241736 | 4.30E-08 | 1.91E-04 | <=0.01 |
| ABI3 | chr17:47280153-47439835 | 1.63E-15 | 1.94E-04 | <=0.01 |
| WTAP | chr6:160100096-160241736 | 4.30E-08 | 2.12E-04 | <=0.01 |
| TET2 | chr4:106067032-106200973 | 1.22E-06 | 2.13E-04 | <=0.01 |
| PIK3CB | chr3:138153428-138553780 | 5.71E-09 | 2.14E-04 | <=0.01 |
| TBXAS1 | chr7:139476850-139763521 | 6.53E-09 | 2.28E-04 | <=0.01 |
| MCU | chr10:73856278-74647452 | 9.40E-06 | 2.29E-04 | <=0.01 |
| CFTR | chr7:117105838-117514193 | 4.17E-08 | 2.29E-04 | <=0.01 |
| FOXL1 | chr16:86612115-86615303 | 1.37E-07 | 2.34E-04 | <=0.01 |
| SUPT6H | chr17:26989302-27188085 | 4.55E-06 | 2.34E-04 | <=0.01 |
| SIPA1 | chr11:65360326-65430565 | 2.29E-11 | 2.55E-04 | <=0.01 |
| PPP2R2A | chr8:26149007-26230196 | 9.65E-07 | 2.68E-04 | <=0.01 |
| IL6R | chr1:154377669-154441926 | 6.69E-15 | 2.69E-04 | <=0.01 |
| MXRA7 | chr17:74671809-74775336 | 3.00E-06 | 2.71E-04 | <=0.01 |
| TOP1 | chr20:39657458-39946312 | 1.12E-08 | 2.75E-04 | <=0.01 |
| STAG1 | chr3:135684515-136729927 | 6.08E-15 | 3.07E-04 | <=0.01 |
| MAP1LC3A | chr20:32868074-33880204 | 6.84E-12 | 3.15E-04 | <=0.01 |
| PLG | chr6:160390131-161695093 | 9.78E-154 | 3.15E-04 | <=0.01 |
| NF2 | chr22:29876181-30127828 | 6.78E-07 | 3.19E-04 | <=0.01 |
| MARK4 | chr19:45715879-45808541 | 5.22E-11 | 3.24E-04 | <=0.01 |
| MYH7B | chr20:32868074-33880204 | 6.84E-12 | 3.41E-04 | <=0.01 |
| SVEP1 | chr9:113127531-113342160 | 1.31E-07 | 3.43E-04 | <=0.01 |
| ARVCF | chr22:19957419-20004331 | 1.08E-06 | 3.44E-04 | <=0.01 |
| PTH1R | chr3:46742823-49894007 | 3.48E-11 | 3.48E-04 | <=0.01 |
| CASZ1 | chr1:10696661-10856707 | 3.72E-06 | 3.64E-04 | <=0.01 |
| NPC1L1 | chr7:44552134-44580914 | 4.36E-06 | 3.71E-04 | <=0.01 |
| RSBN1 | chr1:113933371-114414381 | 6.60E-07 | 3.81E-04 | <=0.01 |
| EHMT1 | chr9:140513444-140764468 | 1.83E-06 | 4.07E-04 | <=0.01 |
| INO80 | chr15:41267889-41775761 | 4.33E-07 | 4.14E-04 | <=0.01 |
| ADRA1A | chr8:26605667-26724790 | 2.13E-06 | 4.19E-04 | <=0.01 |
| LMOD1 | chr1:201592411-201915716 | 1.10E-10 | 4.34E-04 | <=0.01 |
| PLEKHA1 | chr10:124134173-124191867 | 4.22E-06 | 4.37E-04 | <=0.01 |
| RPS6KA1 | chr1:26737269-26901521 | 8.93E-06 | 4.90E-04 | <=0.01 |
| DOK3 | chr5:176910395-176981542 | 1.56E-06 | 4.91E-04 | <=0.01 |
| TRPC4AP | chr20:32868074-33880204 | 6.84E-12 | 5.35E-04 | <=0.01 |
| R3HDM2 | chr12:57643392-57845842 | 4.92E-09 | 5.36E-04 | <=0.01 |
| SERTAD4 | chr1:210111538-210849638 | 2.58E-08 | 5.60E-04 | <=0.01 |
| TIRAP | chr11:126081309-126310239 | 2.00E-07 | 5.67E-04 | <=0.01 |
| CTSK | chr1:150521040-150968110 | 1.29E-09 | 5.69E-04 | <=0.01 |
| VAMP8 | chr2:85766288-85876406 | 1.81E-23 | 5.72E-04 | <=0.01 |
| SCAF11 | chr12:46312914-46385903 | 1.92E-07 | 5.79E-04 | <=0.01 |
| ACVR1B | chr12:52281744-52390862 | 2.73E-06 | 5.92E-04 | <=0.01 |
| NBR1 | chr17:41166622-41466266 | 8.40E-07 | 6.04E-04 | <=0.01 |
| PTPN11 | chr12:111374389-113336686 | 3.72E-27 | 6.36E-04 | <=0.01 |
| SORBS2 | chr4:186506598-186877806 | 6.27E-06 | 6.40E-04 | <=0.01 |
| ZFP64 | chr20:50668202-50820847 | 6.53E-06 | 6.62E-04 | <=0.01 |
| MYOZ2 | chr4:120056939-120550146 | 1.27E-08 | 6.63E-04 | <=0.01 |
| NPEPPS | chr17:45400656-45789427 | 1.69E-06 | 6.93E-04 | <=0.01 |
| PPP2R3A | chr3:135684515-136729927 | 6.08E-15 | 6.99E-04 | <=0.01 |
| ZFPM2 | chr8:106330920-106816760 | 1.88E-08 | 7.10E-04 | <=0.01 |
| SF3B4 | chr1:149895209-149982686 | 1.84E-06 | 7.35E-04 | <=0.01 |
| HIRA | chr22:19318221-19466738 | 8.32E-08 | 7.59E-04 | <=0.01 |
| KDM2B | chr12:121746048-122018920 | 4.74E-06 | 8.12E-04 | <=0.01 |
| P2RY1 | chr3:152552736-152555841 | 2.35E-06 | 8.13E-04 | <=0.01 |
| BCL2 | chr18:60790579-60987361 | 2.43E-06 | 8.79E-04 | <=0.01 |
| SRPR | chr11:126081309-126310239 | 2.00E-07 | 9.05E-04 | <=0.01 |
| NPC1 | chr18:21083462-21166451 | 2.51E-07 | 9.22E-04 | <=0.01 |
| WIPI1 | chr17:66417423-66453653 | 6.60E-07 | 9.98E-04 | <=0.01 |
| HGFAC | chr4:3443614-3451211 | 3.33E-06 | 1.05E-03 | <=0.01 |
| HIVEP2 | chr6:143072604-143266338 | 8.91E-07 | 1.05E-03 | <=0.01 |
| FNDC1 | chr6:159590429-159693141 | 1.33E-08 | 1.06E-03 | <=0.01 |
| PML | chr15:74275547-74339112 | 7.56E-07 | 1.08E-03 | <=0.01 |
| LIN28A | chr1:26737269-26901521 | 8.93E-06 | 1.14E-03 | <=0.01 |
| C5orf13 | chr5:110998318-111755013 | 4.84E-07 | 1.14E-03 | <=0.01 |
| C4orf3 | chr4:120056939-120550146 | 1.27E-08 | 1.16E-03 | <=0.01 |
| PEMT | chr17:17408877-18083116 | 9.51E-10 | 1.17E-03 | <=0.01 |
| C1orf133 | chr1:210111538-210849638 | 2.58E-08 | 1.25E-03 | <=0.01 |
| SEMA5A | chr5:9035138-9550721 | 3.75E-13 | 1.31E-03 | <=0.01 |
| PLEKHG1 | chr6:150920999-151164799 | 6.09E-09 | 1.31E-03 | <=0.01 |
| PSAP | chr10:73576055-73611126 | 7.63E-06 | 1.35E-03 | <=0.01 |
| PGPEP1 | chr19:18451408-18508415 | 1.32E-07 | 1.38E-03 | <=0.01 |
| UNC45A | chr15:91411822-91538859 | 9.86E-27 | 1.41E-03 | <=0.01 |
| BTBD2 | chr19:1905371-2015702 | 6.73E-06 | 1.43E-03 | <=0.01 |
| CELF1 | chr11:47352957-48192393 | 1.90E-06 | 1.44E-03 | <=0.01 |
| RIC8A | chr11:167784-252983 | 8.35E-06 | 1.45E-03 | <=0.01 |
| FAM134C | chr17:40554468-40829048 | 2.33E-07 | 1.48E-03 | <=0.01 |
| MARK3 | chr14:103851701-104003410 | 1.75E-07 | 1.53E-03 | <=0.01 |
| ITIH4 | chr3:52846991-52865495 | 1.22E-08 | 1.56E-03 | <=0.01 |
| ARID4A | chr14:58466453-58840605 | 4.26E-08 | 1.57E-03 | <=0.01 |
| RELB | chr19:45445495-45541452 | 1.53E-06 | 1.62E-03 | <=0.01 |
| FARP1 | chr13:98795434-99102023 | 1.88E-07 | 1.71E-03 | <=0.01 |
| MPZL3 | chr11:118064455-118443685 | 2.01E-06 | 1.72E-03 | <=0.01 |
| FES | chr15:91411822-91538859 | 9.86E-27 | 1.94E-03 | <=0.01 |
| BCAS3 | chr17:58755172-59470198 | 8.51E-10 | 2.09E-03 | <=0.01 |
| TTC39B | chr9:15170843-15307358 | 4.75E-06 | 2.12E-03 | <=0.01 |
| ADD1 | chr4:2845584-3042474 | 5.55E-06 | 2.16E-03 | <=0.01 |
| ABCC10 | chr6:43263432-43418168 | 9.77E-06 | 2.25E-03 | <=0.01 |
| FAM193B | chr5:176910395-176981542 | 1.56E-06 | 2.32E-03 | <=0.01 |
| KCNK5 | chr6:39156749-39197226 | 7.63E-12 | 2.34E-03 | <=0.01 |
| SIK3 | chr11:116706467-117103241 | 4.06E-07 | 2.47E-03 | <=0.01 |
| ATP2B1 | chr12:89813495-90103077 | 8.64E-15 | 2.48E-03 | <=0.01 |
| GMIP | chr19:19322782-19774502 | 3.18E-07 | 2.49E-03 | <=0.01 |
| NAGLU | chr17:40554468-40829048 | 2.33E-07 | 2.52E-03 | <=0.01 |
| SMARCC1 | chr3:46742823-49894007 | 3.48E-11 | 2.69E-03 | <=0.01 |
| UNC13D | chr17:73823310-73975515 | 5.33E-06 | 2.71E-03 | <=0.01 |
| LRRC2 | chr3:46556913-46621589 | 5.39E-06 | 2.75E-03 | <=0.01 |
| RHBDD1 | chr2:227700297-228028829 | 8.24E-06 | 2.92E-03 | <=0.01 |
| ARHGAP26 | chr5:142149949-142608576 | 6.52E-17 | 3.02E-03 | <=0.01 |
| PCDHGB7 | chr5:140710252-141061788 | 4.58E-06 | 3.12E-03 | <=0.01 |
| UBE2Z | chr17:46839593-47045958 | 8.65E-09 | 3.19E-03 | <=0.01 |
| INHBC | chr12:57643392-57845842 | 4.92E-09 | 3.20E-03 | <=0.01 |
| ACTR2 | chr2:65454887-65498387 | 2.40E-07 | 3.25E-03 | <=0.01 |
| PCDHGC3 | chr5:140710252-141061788 | 4.58E-06 | 3.28E-03 | <=0.01 |
| AZGP1 | chr7:99425636-99573780 | 1.70E-06 | 3.28E-03 | <=0.01 |
| ARNTL | chr11:13298199-13408813 | 9.41E-09 | 3.30E-03 | <=0.01 |
| PHOSPHO1 | chr17:47280153-47439835 | 1.63E-15 | 3.49E-03 | <=0.01 |
| MAP3K4 | chr6:160390131-161695093 | 9.78E-154 | 3.56E-03 | <=0.01 |
| NCOA1 | chr2:24714783-25142708 | 5.85E-07 | 3.64E-03 | <=0.01 |
| GOSR1 | chr17:27900487-28853837 | 2.90E-10 | 3.78E-03 | <=0.01 |
| SLC2A12 | chr6:134309835-134373774 | 3.68E-08 | 3.86E-03 | <=0.01 |
| MAU2 | chr19:19322782-19774502 | 3.18E-07 | 3.97E-03 | <=0.01 |
| AEBP2 | chr12:19556979-19873735 | 4.17E-07 | 4.17E-03 | <=0.01 |
| SMARCA4 | chr19:10713121-11172958 | 5.36E-16 | 4.17E-03 | <=0.01 |
| N4BP2L2-IT1 | chr13:32974861-33352157 | 1.19E-10 | 4.20E-03 | <=0.01 |
| RHOA | chr3:46742823-49894007 | 3.48E-11 | 4.21E-03 | <=0.01 |
| KAT2A | chr17:40253422-40307035 | 7.36E-09 | 4.25E-03 | <=0.01 |
| PDS5B | chr13:32974861-33352157 | 1.19E-10 | 4.43E-03 | <=0.01 |
| CCDC92 | chr12:124403207-124457378 | 7.43E-07 | 4.55E-03 | <=0.01 |

**Supplementary Table 11.** Causal genes identified by *TWAS*.

| **chr** | **p0** | **p1** | **assoc genes** | **joint genes** | **best TWAS P** | **best SNP P** | **joint genes** |
| --- | --- | --- | --- | --- | --- | --- | --- |
| 1 | 109098737 | 110517617 | 3 | 1 | 7.00E-10 | 2.90E-10 | PSRC1 |
| 2 | 202952678 | 204788156 | 7 | 1 | 3.90E-11 | 1.80E-11 | ICA1L |
| 3 | 137189600 | 138819532 | 2 | 1 | 4.10E-08 | 3.30E-08 | MRAS |
| 6 | 12020241 | 13990267 | 2 | 1 | 2.40E-13 | 2.50E-11 | RP1-257A7.5 |
| 8 | 19547307 | 20071503 | 1 | 1 | 8.40E-08 | 3.00E-07 | LPL |
| 9 | 21303958 | 22819407 | 2 | 1 | 5.80E-21 | 2.30E-30 | CDKN2B |
| 9 | 135883620 | 136399075 | 1 | 1 | 8.20E-08 | 1.20E-07 | ABO |
| 12 | 111882485 | 112443960 | 1 | 1 | 1.40E-07 | 1.40E-07 | ACAD10 |
| 15 | 78360691 | 79802863 | 2 | 2 | 9.60E-11 | 2.90E-09 | ADAMTS7  MORF4L1 |
| 17 | 46258510 | 47685050 | 1 | 1 | 2.90E-07 | 3.60E-06 | RP11-463M16.4 |
| 19 | 10793749 | 12191233 | 1 | 1 | 4.20E-08 | 9.70E-10 | EPOR |

**Supplementary Table 12.** Topological characteristic of pathways in crosstalk analysis.

| **Pathway** | **Degree** | **Betweenness**  **Centrality** | **Closeness**  **Centrality** | **Average**  **Shortest**  **Path**  **Length** |
| --- | --- | --- | --- | --- |
| Pathways in cancer | 16 | 0.239511 | 0.9 | 1.111111 |
| AGE-RAGE signaling pathway in diabetic complications | 13 | 0.078392 | 0.782609 | 1.277778 |
| Proteoglycans in cancer | 13 | 0.173086 | 0.782609 | 1.277778 |
| Endocytosis | 11 | 0.07016 | 0.72 | 1.388889 |
| PI3K-Akt signaling pathway | 9 | 0.092328 | 0.666667 | 1.5 |
| Focal adhesion | 8 | 0.008777 | 0.642857 | 1.555556 |
| TGF-beta signaling pathway | 8 | 0.001751 | 0.62069 | 1.611111 |
| Colorectal cancer | 8 | 0.001751 | 0.62069 | 1.611111 |
| HTLV-I infection | 8 | 0.001751 | 0.62069 | 1.611111 |
| Chronic myeloid leukemia | 8 | 0.001751 | 0.62069 | 1.611111 |
| Amoebiasis | 7 | 0.002023 | 0.62069 | 1.611111 |
| ECM-receptor interaction | 7 | 0.002023 | 0.62069 | 1.611111 |
| FoxO signaling pathway | 7 | 0 | 0.580645 | 1.722222 |
| Small cell lung cancer | 6 | 0 | 0.580645 | 1.722222 |
| Cytokine-cytokine receptor interaction | 5 | 0.036819 | 0.5625 | 1.777778 |
| Rheumatoid arthritis | 3 | 0 | 0.529412 | 1.888889 |
| Axon guidance | 3 | 0.008831 | 0.529412 | 1.888889 |
| HIF-1 signaling pathway | 2 | 0 | 0.439024 | 2.277778 |
| Phospholipase D signaling pathway | 2 | 0 | 0.461538 | 2.166667 |

“betweenness centrality” defined as the length of shortest paths from all nodes to all other nodes.

“degree” of a node represented the number of edges linked to that node.

**Supplementary Table 13.** eSNPs identified in Sherlock and SMR using blood that are also eQTLs in CAD-related tissues.

| **SNP** | **Methods** | **Blood eQTL for** | **Blood eQTL *P*value** | **Adipose eQTL for** | **Adipose eQTL *P*value** | **Liver eQTL for** | **Liver eQTL *P*value** | **Muscle skeletal eQTL for** | **Muscle skeletal eQTL *P*value** | **Artery eQTL for** | **Artery eQTL *P*value** | **Brain eQTL for** | **Brain eQTL *P*value** |
| --- | --- | --- | --- | --- | --- | --- | --- | --- | --- | --- | --- | --- | --- |
| rs646776 | Sherlock | PSRC1 | 9.38E-09 | PSRC1 | 1.30E-06 | CELSR2 | 4.70E-29 | CELSR2 | 7.00E-69 |  |  | PSRC1 | 9.50E-10 |
|  |  |  |  |  |  | SORT1 | 7.00E-46 | PSRC1 | 4.80E-14 |  |  |  |  |
|  |  |  |  |  |  | PSRC1 | 4.80E-33 |  |  |  |  |  |  |
|  |  |  |  |  |  | ATXN7L2 | 2.90E-06 |  |  |  |  |  |  |
|  |  |  |  |  |  | SYPL2 | 4.50E-06 |  |  |  |  |  |  |
| rs12217501 | Sherlock | NT5C2 | 3.73E-07 | MARCKSL1P1 | 1.30E-09 |  |  | ARL3 | 1.70E-05 | MARCKSL1P1 | 4.10E-07 | ARL3 | 2.70E-06 |
|  |  |  |  | ARL3 | 1.80E-05 |  |  | WBP1L | 3.80E-04 | CALHM2 | 7.90E-06 | CNNM2 | 1.70E-05 |
|  |  |  |  | CALHM2 | 2.80E-05 |  |  |  |  | NT5C2 | 1.40E-06 | NT5C2 | 5.90E-05 |
|  |  |  |  | PDCD11 | 9.90E-05 |  |  |  |  | WBP1L | 2.40E-04 |  |  |
|  |  |  |  | SFXN2 | 2.60E-04 |  |  |  |  | ARL3 | 3.90E-04 |  |  |
| rs7276739 | Sherlock | MRPS6 | 6.50E-06 |  |  |  |  |  |  |  |  |  |  |
| rs7579665 | Sherlock | MAT2A | 1.18E-06 | GGCX | 1.80E-04 |  |  | C2orf68 | 4.50E-05 | GGCX | 3.40E-06 | GGCX | 3.50E-08 |
| rs2246942 | Sherlock | LIPA | 4.84E-34 | LIPA | 4.90E-16 |  |  | LIPA | 6.50E-11 | LIPA | 1.30E-05 | LIPA | 2.10E-07 |
| rs1894400 | Sherlock | FES | 1.03E-08 | FES | 3.10E-15 |  |  |  |  | FES | 5.90E-15 |  |  |
|  |  |  |  |  |  |  |  |  |  | MAN2A2 | 3.20E-05 |  |  |
| rs7568458 | Sherlock | GGCX | 3.09E-32 | GGCX | 8.60E-18 |  |  | GGCX | 6.40E-22 | GGCX | 4.50E-34 | GGCX | 1.40E-38 |
|  |  |  |  | VAMP8 | 9.10E-06 |  |  | VAMP5 | 2.30E-07 | MAT2A | 3.40E-06 | POLR1A | 8.20E-05 |
| rs11206510 | Sherlock | BSND | 4.53E-06 |  |  |  |  |  |  |  |  |  |  |
| rs2068814 | Sherlock | FAM177B | 6.88E-11 | MIA3 | 8.70E-06 |  |  | AIDA | 5.80E-05 |  |  | MIA3 | 1.70E-06 |
|  |  |  |  |  |  |  |  |  |  |  |  | AIDA | 3.80E-12 |
| rs2166529 | Sherlock | USP39 | 2.53E-06 | GGCX | 7.60E-14 |  |  | GGCX | 4.60E-19 | GGCX | 4.40E-26 | GGCX | 2.90E-32 |
|  |  |  |  | VAMP8 | 1.40E-07 |  |  | VAMP5 | 1.70E-07 | MAT2A | 4.30E-06 |  |  |
|  |  |  |  |  |  |  |  | USP39 | 5.50E-05 |  |  |  |  |
| rs5760229 | Sherlock | CABIN1 | 7.26E-08 | SUSD2 | 5.80E-32 | SUSD2 | 1.40E-12 | SUSD2 | 1.10E-27 | SUSD2 | 3.60E-09 | CABIN1 | 1.80E-05 |
|  |  |  |  |  |  |  |  |  |  |  |  | SUSD2 | 1.80E-09 |
| rs635634 | Sherlock | ABO | 5.81E-15 | ABO | 3.40E-24 | ABO | 3.60E-09 | ABO | 9.20E-18 | ABO | 7.70E-16 | SURF1 | 1.80E-07 |
|  |  |  |  | SURF1 | 2.20E-10 |  |  | SURF1 | 1.60E-08 | SURF1 | 1.10E-06 |  |  |
| rs8141797 | Sherlock | SUSD2 | 7.89E-07 | SUSD2 | 1.30E-32 | SUSD2 | 4.10E-10 | SUSD2 | 1.80E-26 | SUSD2 | 4.70E-09 | CABIN1 | 3.90E-05 |
|  |  |  |  |  |  |  |  |  |  |  |  | SUSD2 | 2.00E-09 |
| rs10096633 | Sherlock | LPL | 8.95E-06 |  |  |  |  |  |  |  |  |  |  |
| rs10485505 | Sherlock | EIF6 | 1.47E-06 | EIF6 | 1.00E-05 |  |  | EIF6 | 1.40E-09 | EDEM2 | 2.70E-04 |  |  |
|  |  |  |  | MMP24-AS1 | 8.40E-05 |  |  | PROCR | 3.60E-06 |  |  |  |  |
|  |  |  |  | PROCR | 1.50E-04 |  |  |  |  |  |  |  |  |
|  |  |  |  | EDEM2 | 3.00E-04 |  |  |  |  |  |  |  |  |
| rs11105325 | Sherlock | GALNT4 | 7.75E-06 | POC1B | 6.00E-14 | POC1B | 3.50E-06 |  |  | POC1B | 4.80E-13 | POC1B | 2.10E-16 |
| rs7528419 | SMR | PSRC1 | 3.40E-64 | PSRC1 | 1.20E-06 | SORT1 | 3.10E-49 | CELSR2 | 2.20E-76 |  |  | PSRC1 | 6.20E-09 |
|  |  |  |  |  |  | PSRC1 | 2.40E-35 | PSRC1 | 8.70E-13 |  |  |  |  |
|  |  |  |  |  |  | CELSR2 | 1.00E-32 |  |  |  |  |  |  |
|  |  |  |  |  |  | ATXN7L2 | 5.80E-07 |  |  |  |  |  |  |
|  |  |  |  |  |  | SYPL2 | 6.50E-07 |  |  |  |  |  |  |
| rs1412444 | SMR | LIPA | 2.28E-276 | LIPA | 2.00E-10 |  |  | LIPA | 1.50E-11 | LIPA | 5.80E-18 | LIPA | 2.40E-07 |
| rs173396 | SMR | SWAP70 | 1.84E-69 |  |  |  |  |  |  | SWAP70 | 2.70E-11 | ARL3 | 1.70E-05 |
| rs11191607 | SMR | NT5C2 | 1.04E-49 | MARCKSL1P1 | 2.00E-09 |  |  | ARL3 | 3.60E-05 | CALHM2 | 4.20E-05 | AS3MT | 1.60E-04 |
|  |  |  |  | ARL3 | 8.70E-06 |  |  |  |  | MARCKSL1P1 | 1.70E-06 | CNNM2 | 2.20E-05 |
|  |  |  |  | CALHM2 | 2.10E-05 |  |  |  |  | NT5C2 | 5.40E-07 |  |  |
|  |  |  |  | PDCD11 | 1.70E-04 |  |  |  |  |  |  |  |  |
|  |  |  |  | SFXN2 | 2.10E-04 |  |  |  |  |  |  |  |  |
| rs2366639 | SMR | VAMP8 | 9.23E-196 | GGCX | 1.30E-16 |  |  | GGCX | 1.30E-17 | GGCX | 1.20E-31 | GGCX | 5.00E-34 |
|  |  |  |  | VAMP8 | 3.50E-05 |  |  | VAMP5 | 1.10E-04 |  |  |  |  |
| rs7579665 | SMR | MAT2A | 8.13E-14 | GGCX | 1.80E-04 |  |  | C2orf68 | 4.50E-05 | GGCX | 3.40E-06 | GGCX | 3.50E-08 |
